# Simple and robust diagnosis of early, small and AFP-negative primary hepatic carcinomas: an integrative approach of serum fluorescence and conventional blood tests

**DOI:** 10.18632/oncotarget.11771

**Published:** 2016-08-31

**Authors:** Ting Wang, Kun-He Zhang, Piao-Ping Hu, Zeng-Yong Huang, Pan Zhang, Qin-Si Wan, De-Qiang Huang, Nong-Hua Lv

**Affiliations:** ^1^ Department of Gastroenterology, The First Affiliated Hospital of Nanchang University, Jiangxi Institute of Gastroenterology and Hepatology, Nanchang 330006, China

**Keywords:** primary hepatic carcinoma, early diagnosis, AFP-negative hepatoma, diagnostic model, serum fluorescence

## Abstract

The diagnosis of early, small and alpha-fetoprotein (AFP)-negative primary hepatic carcinomas (PHCs) remains a significant challenge. We developed a simple and robust approach to noninvasively detect these PHCs. A rapid, high-throughput and single-tube method was firstly developed to measure serum autofluorescence and cell-free DNA (cfDNA)-related fluorescence using a real-time PCR system, and both types of serum fluorescence were measured and routine laboratory data were collected in 1229 subjects, including 353 PHC patients, 331 liver cirrhosis (LC) patients, 213 chronic hepatitis (CH) patients and 332 normal controls (NC). The results showed that fluorescence indicators of PHC differed from those of NC, CH and LC to various extents, and all of them were not associated with age, gender, or AFP level. The logistic regression models established with the fluorescence indicators alone and combined with AFP, hepatic function tests and blood cell analyses were valuable for distinguishing early, small, AFP-negative and all PHC from LC, CH, NC and all non-PHC, with areas under the receiver operating characteristic curves 0.857–0.993 and diagnostic accuracies 80.2–97.7%. Conclusively, serum autofluorescence and cfDNA-related fluorescence are able to be rapidly and simultaneously measured by our simple method and valuable for diagnosing early, small and AFP-negative PHCs, especially integrating with AFP and conventional blood tests.

## INTRODUCTION

Primary hepatic carcinoma (PHC), including hepatocellular carcinoma (HCC) and intrahepatic cholangiocarcinoma (ICC), is one of the most common malignant tumors worldwide. The incidence and mortality of PHC have increased in recent decades, whereas they have been declining for most cancers [1, 2]. The survival of PHC patients strongly depends on the tumor stage at the time of diagnosis according to Barcelona Clinic Liver Cancer (BCLC) staging classification [3]. Therefore, providing a diagnosis and treatment as early as possible is crucial for improving the prognosis of PHC patients.

Alpha-fetoprotein (AFP) has been the most widely used serum biomarker for PHC diagnosis. Unfortunately, nearly 40% of PHC patients are AFP-negative (< 20 ng/mL), especially patients harboring early or small PHCs [4, 5]. In recent decades, numerous efforts have been made to discover new biomarkers that overcome the shortfalls of AFP, which has led to the identification of several potential biomarkers [6], such as Golgi protein 73 (GP73) [7], glypican-3 (GPC-3) [8], and microRNAs [9]. However, inadequate sensitivity and/or specificity for diagnosing PHC limit the application of these markers in clinical practice [7–9], and the diagnosis of early, small and AFP-negative PHCs remains a significant challenge.

A few reports have shown that the serum autofluorescence produced by native fluorescent substances in the blood may be used to diagnose liver cancer [10–12]. The laser-induced serum Raman spectra of patients with liver cancer differed from those of cirrhosis patients or normal subjects [11], and this difference was diagnostically significant for liver cancer [10]. However, the principle component of the spectra could not discriminate cirrhosis patients with and without HCC [12]. Moreover, these studies all examined small samples and did not evaluate the early detection of liver cancer; therefore, further systematic evaluations are warranted.

Circulating cell-free DNA (cfDNA) has garnered considerable attention as a potential cancer biomarker. It was complementary to current tumor markers in diagnosis because its level did not correlate with the concentrations of the tumor markers [13]. Serum cfDNA levels are significantly higher in HCC patients than in control subjects and are associated with tumor size and differentiation but not age, gender, TNM stage or the levels of AFP or des-γ-carboxy prothrombin (DCP) [14]. A recent meta-analysis showed that cfDNA is more valuable for diagnosing HCC than AFP [15], but the use of cfDNA alone is not recommended for HCC diagnosis due to its non-robust performance.

It is well known that the sensitivity and/or specificity of a single biomarker for tumor diagnosis are invariably inadequate. Thus, the combination of several biomarkers for diagnosis is a reasonable strategy by which to address this problem [11]. Interestingly, the combination of conventional blood tests is useful for predicting [16] and diagnosing [17] HCC, especially when combined with AFP. Therefore, the combination of specific tests with conventional laboratory blood tests to develop diagnostic models is attractive in current diagnostics.

In the present study, we firstly developed a simple method using a conventional real-time PCR system to measure the serum autofluorescence and cfDNA-related fluorescence in PHC, liver cirrhosis (LC), chronic hepatitis (CH) patients and normal control (NC) subjects, and then systematically evaluated the diagnostic significance of both types of serum fluorescence alone and combined with AFP, hepatic function tests and/or conventional laboratory blood tests for diagnosing PHC, particularly early (BCLC stage A), small (tumor size ≤ 3 cm) and AFP-negative PHCs.

## RESULTS

### Demographic and clinical data of subjects

A total of 1229 subjects were entered into this study, including 353 PHC patients, 331 LC patients, 213 CH patients and 332 NC subjects. The demographic and clinical characteristics of these subjects are shown in Table 1.

**Table 1:** Demographic and clinical characteristics of the subjects

	PHC (*n*=353)	LC (*n* = 331)	CH (*n* = 213)	NC (*n*= 332)	*P*
Age (mean ± SD, years)	52.9 ± 12.9	50.8 ± 12.0*	36.7 ± 12.7**	47.0 ± 17.6**	< 0.01^a^
Gender [n (%)]					
Male	290 (82.2)	260 (78.5)	166 (77.9)	184 (55.4)	< 0.001^b^
Female	63(17.8)	71(21.5)	47(22.1)	148 (44.6)	
Etiology [n (%)]					
HBV	305 (86.4)	263 (79.5)	200 (93.9)	-	< 0.001^b^
HCV	0 (0.0)	4 (1.2)	12 (5.6)	-	< 0.001^c^
Alcohol	4 (1.1)	10 (3.0)	0 (0.0)	-	0.001^c^
Schistosomiasis	0 (0.0)	3 (0.9)	0 (0.0)	-	0.043^c^
Mixed	11(3.0)	39 (11.8)	1 (0.5)	-	< 0.001^c^
Unknown	33 (9.4)	12 (3.6)	0 (0.0)	-	< 0.001^c^
AFP (μg/L)	367.6 ± 517.7	12.8 ± 50.6**	63.6 ± 163.0**	2.2 ± 1.1**	< 0.01^a^
Hepatic function tests (mean ± SD)					
ALT(U/L)	60.2 ± 95.7	54.9 ± 173.6	262.8 ± 259.0**	19.2 ± 9.2**	< 0.01^a^
AST(U/L)	86.6 ± 131.1	62.7 ± 116.4**	148.4 ± 158.0**	22.5 ± 4.9**	< 0.01^a^
TBIL(μmol/L)	29.5 ± 49.7	38.0 ± 71.2	59.2 ± 94.4**	10.1 ± 3.6**	< 0.01^a^
DBIL(μmol/L)	17.8 ± 35.4	21.9 ± 45.0	38.6 ± 68.6**	4.3 ± 1.4**	< 0.01^a^
TP(g/L)	65.5 ± 6.9	61.0 ± 9.3**	65.7 ± 7.2	73.9 ± 3.6**	<0.01^a^
ALB(g/L)	36.1 ± 6.0	31.7 ± 6.0**	38.1 ± 5.6**	47.1 ± 2.8**	< 0.01^a^
GLB(g/L)	29.4 ± 6.2	29.4 ± 7.8	27.6 ± 5.2**	26.8 ± 2.9**	< 0.01^a^
Child-Pugh grade [n (%)]					
A	284 (80.5)	174 (52.6)	141 (66.2)	-	< 0.01^b^
B	41 (11.6)	65 (19.6)	42 (19.7)	-	
C	28 (7.9)	92 (27.8)	30 (14.1)	-	
Blood cell analyses					
WBC(10^9^/L)	6.3 ± 3.6	4.3 ± 2.6**	5.4 ± 1.9**	6.3 ± 1.5	< 0.001^a^
RBC(10^12^/L)	4.1 ± 0.9	3.5 ± 0.9**	4.5 ± 0.7**	4.8 ± 0.5**	< 0.001^a^
Hb(g/L)	121.0 ± 24.5	98.4 ± 25.2**	134.9 ± 19.8**	141.9 ± 13.2**	< 0.001^a^
PLT(10^9^/L)	157.3 ± 92.1	91.2 ± 76.0**	163.1 ± 66.5	218.3 ± 49.9**	< 0.001^a^

Note:

* *P* < 0.05,

** *P* < 0.01, compared with PHC.

a One-way ANOVA test;

b Pearson chi-squared test;

c Fisher's exact test.

PHC: primary hepatic carcinoma; LC: liver cirrhosis; CH: chronic hepatitis; NC: normal control; HBV: hepatitis B virus; HCV: hepatitis C virus; AFP: alpha-fetoprotein; ALT: alanine aminotransaminase; AST: aspartate aminotransaminase; TBIL: total serum bilirubin; DBIL: direct serum bilirubin; TP: total serum protein; ALB: serum albumin; GLB: serum gamma-globins; WBC: white blood cell; RBC: red blood cell; Hb: hemoglobin; PLT: platelet.

### Optimum conditions for the measurement of PHC serum fluorescence intensity

**Figure 1:**
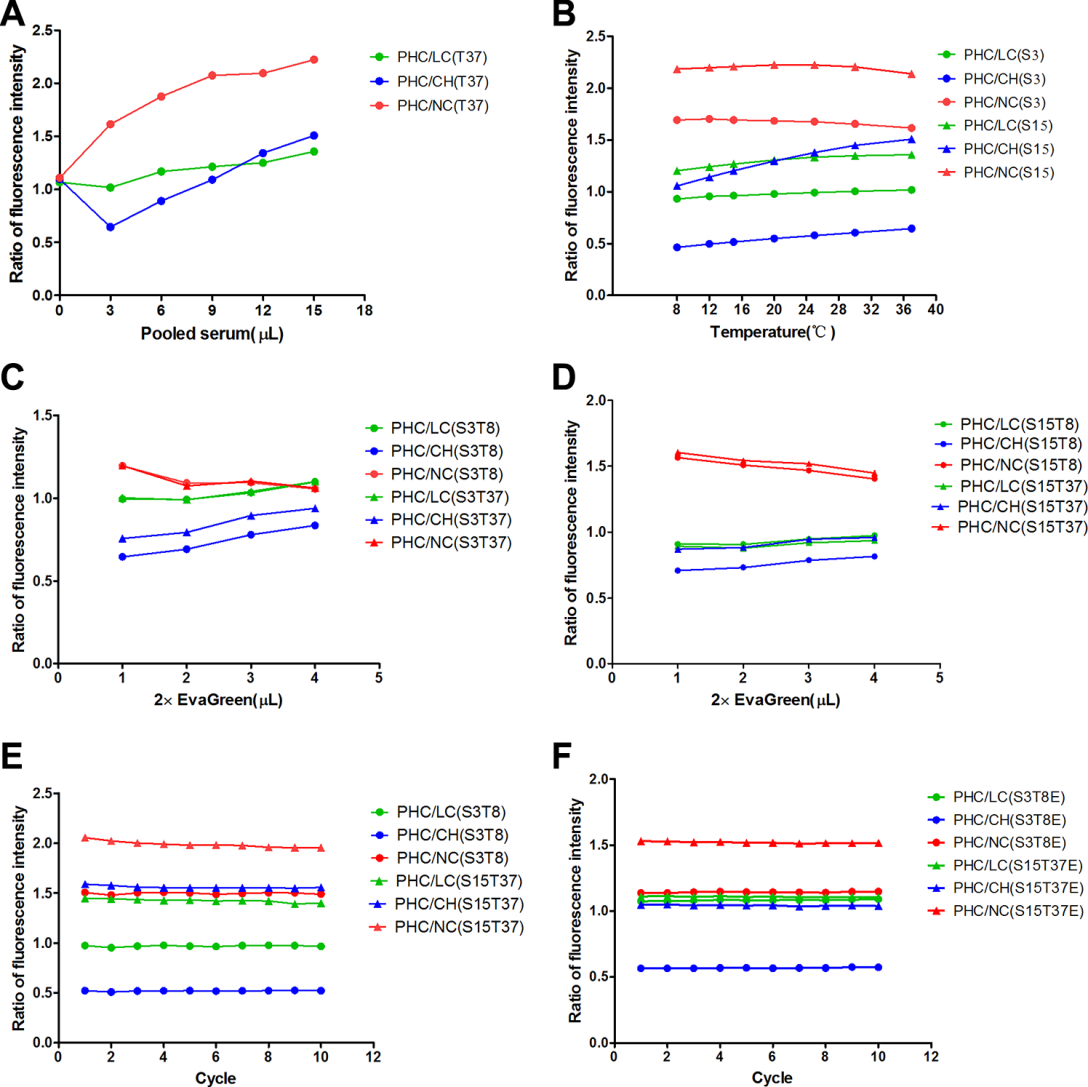
Optimum conditions for the fluorescence intensity measurements of pooled serum samples (**A**) The fluorescence intensity ratios of PHC to LC, CH and NC correspond to pooled serum volumes at 37°C (T37). (**B**) The fluorescence intensity ratios of PHC to LC, CH and NC correspond to detection temperatures for 3 μL (S3) and 15 μL (S15) of pooled serum. (**C**, **D**) The fluorescence intensity ratios of PHC to LC, CH and NC correspond to EvaGreen volumes for 3 μL (S3) and 15 μL (S15) of pooled serum at 8°C (T8) and 37°C (T37). (**E**, **F**) The fluorescence intensity ratios of PHC to LC, CH and NC correspond to the cycle numbers (incubation time) for 3 μL of pooled serum at 8°C (S3T8) and 15 μL of pooled serum at 37°C (S15T37) in the absence of EvaGreen and for 3 μL of pooled serum at 8°C (S3T8E) and 15 μL of pooled serum at 37°C (S15T37E) in the presence of EvaGreen. PHC: primary hepatic carcinoma; LC: liver cirrhosis; CH: chronic hepatitis; NC: normal control.

All fluorescence intensity (FI) ratios of PHC to LC, CH and NC samples directly correlated with the pooled serum volume and stabilized at 15 μL (Figure 1A). The optimum serum volumes were 3 μL for differentiating PHC from CH and 15 μL for differentiating PHC from LC and NC.

The FI ratios of PHC to LC and CH slightly increased as the detection temperature increased in either 3 μL or 15 μL serum samples, but the ratios of PHC to NC slightly decreased (Figure 1B). The optimum temperatures were 8°C for differentiating PHC from CH (at 3 μL) and NC and 37°C for differentiating PHC from CH (at 15 μL) and LC.

The amount of EvaGreen positively correlated with the FI ratios of PHC to LC and CH but negatively correlated with the ratio of PHC to NC (Figure 1C, 1D). A volume of 3 μL was selected as the optimum volume of EvaGreen because it balanced the values for differentiating PHC from LC, CH and NC.

The number of cycles (incubation time) did not affect the FI ratios of PHC to LC, CH and NC; thus, the first cycle was selected for analyses (Figure 1E, 1F).

### The fluorescence intensity measured in serum specimens

**Figure 2:**
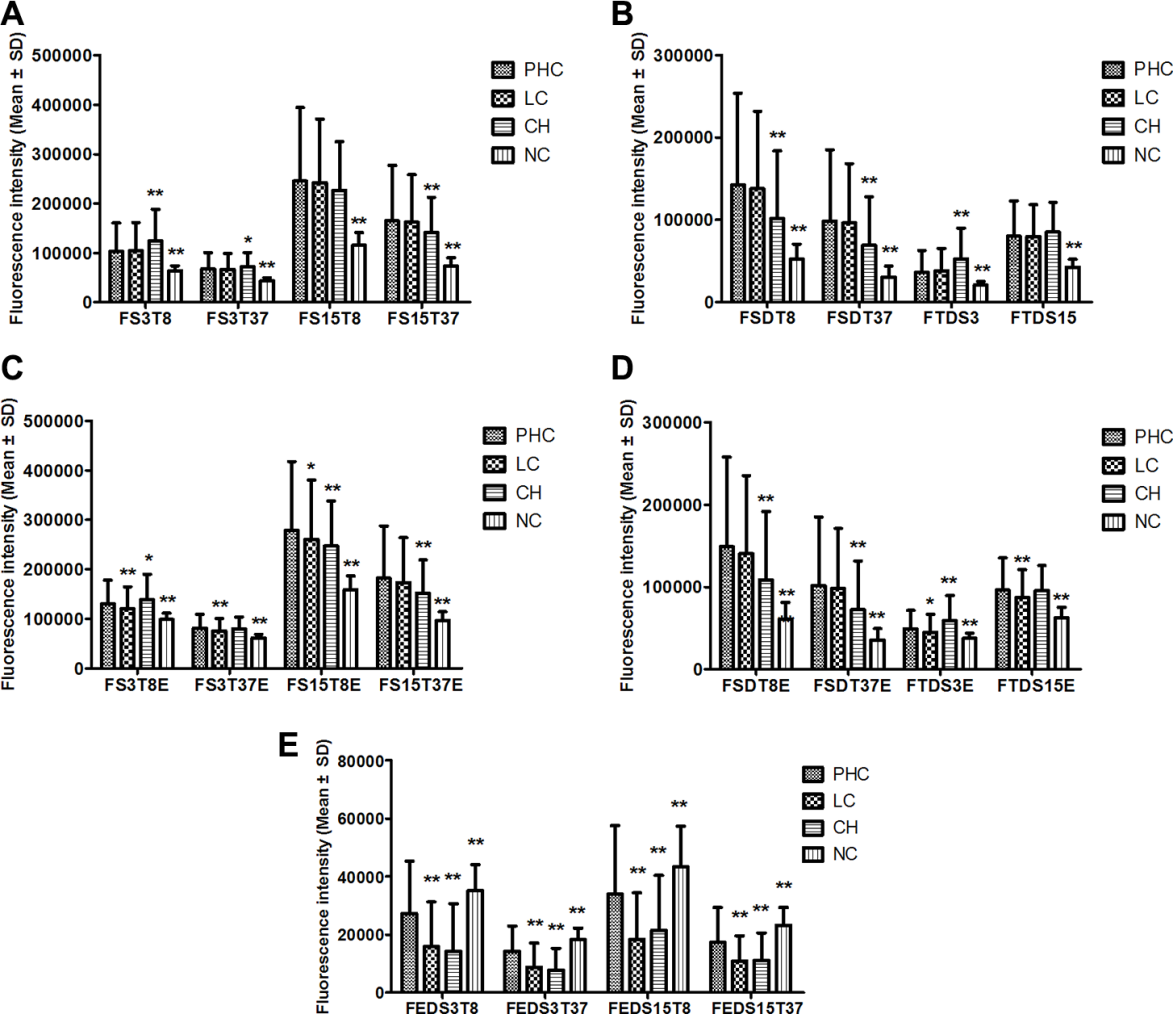
The serum fluorescence intensities of the PHC, LC, CH and NC groups (**A**–**E**) The comparisons of different sub-groups of fluorescence indicators between PHC and LC, CH or NC. *P < 0.05, **P < 0.01, compared with the PHC group by multiple comparisons (Dunnett's T3 test) after ANOVA. PHC: primary hepatic carcinoma; LC: liver cirrhosis; CH: chronic hepatitis; NC: normal control. The names of the fluorescence indicators are combinations of several abbreviations representing the fluorescence intensity (F) of 3 μL (S3) or 15 μL (S15) of serum at a detection temperature of 8°C (T8) or 37°C (T37) in the presence of EvaGreen (**E**). Moreover, the names indicate differences for two indicators between 3 μL and 15 μL (SD) of serum, for two temperatures of 8°C and 37°C (TD) and for the presence or absence of EvaGreen (ED).

The fluorescence intensities of the four groups are shown in Figure 2. All FI indicators in the NC group and most FI indicators in the CH group significantly differed from those of the PHC group, but only the indicators related to EvaGreen significantly differed between the LC and the PHC groups.

**Figure 3:**
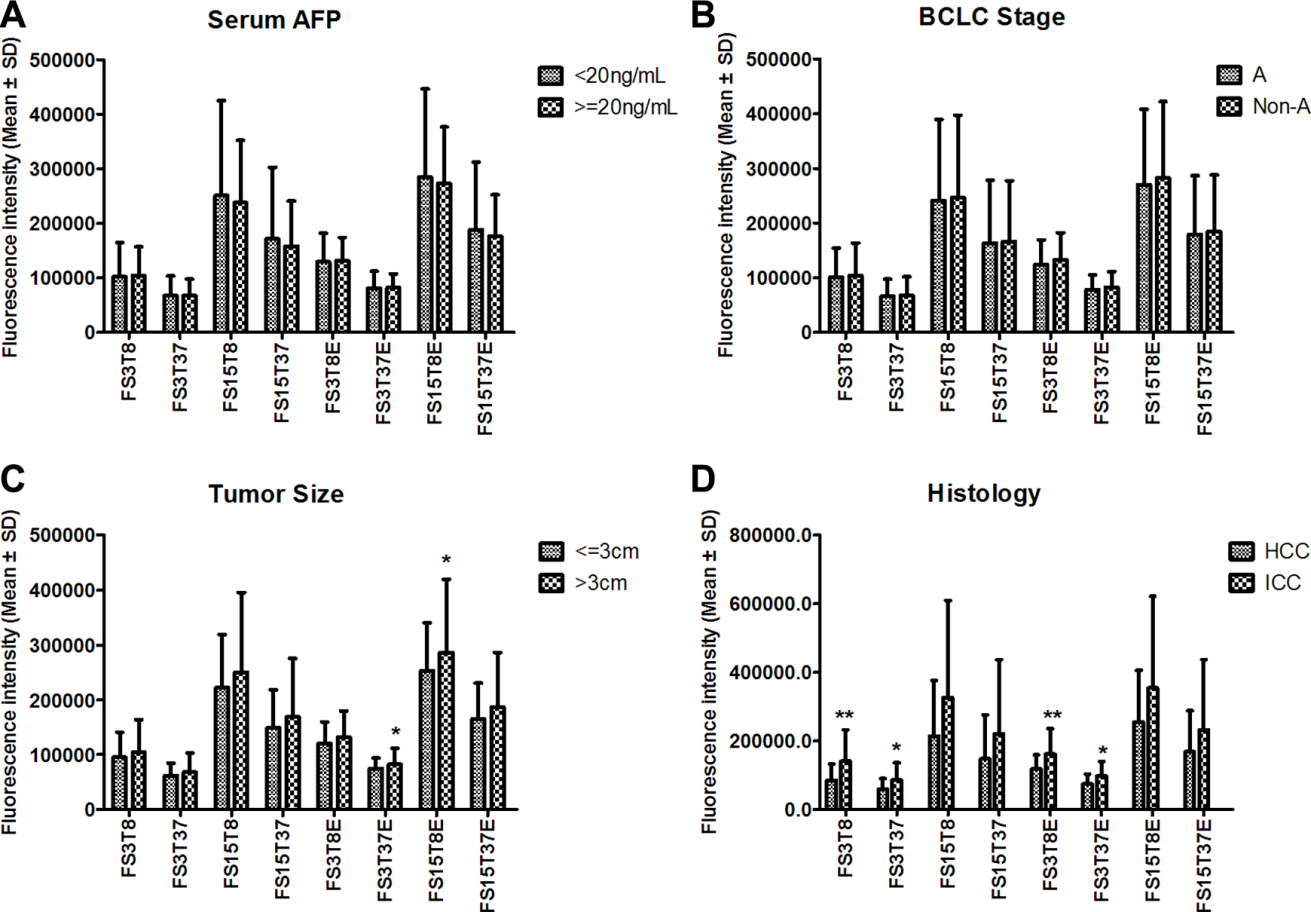
The comparisons of 8 original fluorescence indicators between PHC sub-groups based on serum AFP level (**A**), BCLC stage (**B**), tumor size (**C**), and tumor histology (**D**). *P < 0.05, **P < 0.01, compared between two subgroups. AFP: alpha-fetoprotein; BCLC: Barcelona Clinic Liver Cancer stage; HCC: hepatocellular carcinoma; ICC: intrahepatic cholangiocarcinoma; PHC: primary hepatic carcinoma. The name of each fluorescence indicator is a combination of several abbreviations representing the fluorescence intensity (F) of 3 μL (S3) or 15 μL (S15) of serum at a detection temperature of 8°C (T8) or 37°C (T37) in the presence (E) or absence of EvaGreen.

The PHC patients were sub-grouped by serum AFP level, BCLC stage, tumor size and histological type. We compared 8 original fluorescence indicators between subgroups and found that none of the indicators significantly differed between the AFP-negative (*n* = 193) and AFP-positive (*n* = 160) subgroups (Figure 3A) or between the BCLC stage A (*n* = 99) and non-A (*n* = 254) subgroups (Figure 3B). Only two indicators significantly differed between the small (*n* = 67) and large (*n* = 225) subgroups (Figure 3C) (292 PHC cases with exact tumor size). These results suggest that neither the serum autofluorescence nor the cfDNA-related fluorescence is associated with AFP or the BCLC stage and that the cfDNA-related fluorescence is slightly associated with tumor size. However, the serum fluorescence was higher in the ICC subgroup (*n* = 27) than in the HCC subgroup (*n* = 69), and four indicators significantly differed between the subgroups (Figure 3D).

### The association of serum fluorescence intensity with age and gender

The original FI indicators were almost not significantly different between the PHC and LC groups but mostly significantly different between the PHC and CH groups and all significantly different between PHC and NC groups. These significant differences may have resulted from the significant differences in age between the PHC and CH groups and in gender ratios between the PHC and NC groups (Table 1). To clarify the effects of age and gender on the serum fluorescence intensity, we used a binary logistic regression analysis to calculate the crude odds ratio (OR) and the adjusted OR for age and gender (ORa) of each original indicator with significant OR for PHC vs. LC, CH, NC and NPHC (NC+LC+CH) and its correlation coefficients with age (Ra) and gender (Rg). The results showed that the serum FI is generally independent of age and gender (Table S1).

### Correlations of serum fluorescence intensity with routine laboratory blood test results

Although the serum FI was generally not related to age or gender, it might be related to laboratory blood test results. Therefore, the correlations of 8 FI indicators with AFP, AST, ALT, TBIL, DBIL, IDIL, TP, ALB, GLB, WBC, RBC, Hb, and PLT were analyzed with Pearson correlation analysis. Of 104 correlation coefficients (absolute value), 23 (22.1%) were < 0.099, 59 (56.7%) ranged from 0.101–0.291, 17 (16.3%) ranged from 0.315–0.492 and 5 (4.8%) ranged from 0.510–0.530 (Table S2). All 8 FI indicators negatively and moderately correlated with albumin levels. The FI indicators of 3 μL serum samples positively and moderately correlated with serum TBIL, DBIL and IBIL levels, whereas the other indicators did not correlate or weakly correlated with these laboratory blood test results.

### Diagnostic value of single indicators of fluorescence intensity and conventional blood tests for PHC

All 20 serum FI indicators significantly differentiated PHC from NC, especially the indicators of the 15 mL serum samples, with the areas under the receiver operating characteristic (ROC) curve (AUROCs) of 0.648–0.925; 16 indicators significantly differentiated PHC from CH, with AUROCs of 0.563–0.727; 9 indicators significantly differentiated PHC from LC (mainly indicators related to EvaGreen), with AUROCs of 0.544–0.712; 19 indicators significantly differentiated PHC from NPHC, with AUROCs of 0.549–0.696. All 4 difference indicators between the presence and absence of EvaGreen significantly differentiated PHC from NC, LC, CH or NPHC. Forty-four of the 48 indicators of AFP, hepatic function tests and blood cell analyses were significant for differentiating PHC from NC, LC, CH or NPHC, with AUROCs of 0.546–0.958. See Table S3 for more details.

Importantly, the indicators, either in the serum fluorescence or in laboratory blood test results, exhibited different AUROCs in discriminating PHC from various control groups, indicating that these indicators complement each other for PHC diagnosis.

### The establishment and evaluation of diagnostic models for PHC

The subjects were randomly divided into the training set (approximately 80% of all cases) and the validation set (the other cases). The training set was used to establish diagnostic models for discriminating PHC from NC, LC, CH or NPHC by applying a multinomial logistic stepwise regression analysis in which the covariates were indicators of the serum FI, AFP, hepatic function tests and blood cell analyses alone or in different combinations. In the models combining serum fluorescence with laboratory blood test results, only 8 original FI indicators were used to ensure a reasonable modeling algorithm and fewer variables in the models. Eight groups of models were established (Table 2). The models were named based on a combination of abbreviations representing the fluorescence intensity (F), alpha-fetoprotein (A), hepatic function tests (H) and/or blood cell analyses (B) with model (−M), such as FAHB-M (model established with the indicators of fluorescence intensity, alpha-fetoprotein, hepatic function tests and blood cell analyses). All models were significant (*P* < 0.001), with a Nagelkerke Pseudo R^2^ ranging from 0.321 to 0.909. The combination of FI with any laboratory blood test result increased the Pseudo R^2^.

**Table 2:** Main information of various diagnostic models for PHC

Model	Variables in model#	Nagelkerke Pseudo R^2^	Likelihood ratio test	
			χ^2^	*P*
F-M	FTDS15E, FS15T8, FS3T8E, FS3T8, FS3T37, FS3T37E, FS15T37, FTDS3, FTDS15, FEDS3T37	0.711	1062.7	0.000
A-M	AFP	0.321	356.2	0.000
H-M	TBIL, ALT, AST, DBIL, ALB	0.814	1409.4	0.000
B-M	PLT, WBC, Hb	0.552	700.6	0.000
FA-M	AFP, FS15T37E, FS15T37, FS3T37E, FS3T8, FS3T8E, FS15T8, FS15T8E	0.737	1169.3	0.000
FH-M	TBIL, ALT, FS15T37E, FS3T37E, FS3T8, FS15T8, FS15T8E, AST, DBIL, ALB	0.877	1719.2	0.000
FB-M	PLT, RBC, FS15T8E, FS15T8, FS3T8E, FS3T8, FS3T37E, FS15T37, WBC, Hb	0.795	1362.6	0.000
FAHB-M	PLT, ALT, FS15T8E, FS15T8, FS3T8, FS3T37E, FS15T37, AFP, AST, ALB, WBC, Hb	0.909	1857.4	0.000

Note: #The variables in the models were transformed with a natural logarithm. Each model name is a combination of abbreviations representing the fluorescence intensity (F), alpha-fetoprotein (A), hepatic function tests (H) and/or blood cell analyses (B) with the model (−M), indicating the covariates used during modeling; for example, FAHB-M was established with the indicators of fluorescence intensity, alpha-fetoprotein, hepatic function tests and blood cell analyses. The fluorescence indicators represent the fluorescence intensity (F) of 3 μL (S3) or 15 μL (S15) serum samples at a detection temperature of 8°C (T8) or 37°C (T37) in the presence (E) or absence of EvaGreen. AFP: alpha-fetoprotein, ALT: alanine transaminase; AST: aspartate transaminase; TBIL: total serum bilirubin; DBIL: direct serum bilirubin; ALB: serum albumin; WBC: white blood cell; RBC: red blood cell; Hb: hemoglobin; PLT: platelet.

The AUROCs and diagnostic performances of the models established with serum fluorescence indicators alone (F-M) and combined with AFP, hepatic function tests and blood cell analyses (FAHB-M) are shown in Table 3. Both models yielded similar AUROCs and diagnostic performances for the training and validation sets. The F-M model was excellent in discriminating PHC from NC (AUROC ≥ 0.95) and good or fair in discriminating PHC from LC and CH (AUROCs 0.764–0.810). The FAHB-M model was excellent in discriminating PHC from NC, LC, CH or NPHC (AUROCs 0.901–0.995).

**Table 3:** Diagnostic value of models F-M and FAHB-M for PHC

	F-M		FAHB-M	
	Training set	Validation set	Training set	Validation set
PHC vs. NC (n/n)	268/272	85/60	280/258	73/74
AUROC(95%CI)	0.963 (0.947-0.978)	0.972 (0.951-0.994)	0.993 (0.987-0.999)	0.995 (0.987-1.000)
Sensitivity (%)	90.7	91.8	97.4	96.5
Specificity (%)	92.6	91.7	97.4	100.0
Accuracy (%)	91.7	91.7	97.4	97.9
PPV/NPV (%)	92.4/91.0	94.0/88.7	97.4/97.4	100.0/95.2
PLR/NLR	12.33/0.10	11.01/0.09	37.84/0.03	−/0.04
PHC vs. LC (n/n)	268/263	85/68	280/268	73/63
AUROC(95%CI)	0.777 (0.738-0.816)	0.764 (0.690-0.838)	0.919 (0.895-0.943)	0.901 (0.853-0.950)
Sensitivity (%)	70.1	71.8	82.1	85.9
Specificity (%)	74.1	67.6	89.4	82.4
Accuracy (%)	72.1	69.9	85.7	84.3
PPV/NPV (%)	73.4/70.9	73.5/65.7	88.7/83.0	85.9/82.4
PLR/NLR	2.71/0.40	2.22/0.42	7.71/0.20	4.87/0.17
PHC vs. CH (n/n)	268/168	85/45	280/173	73/40
AUROC(95%CI)	0.797 (0.755-0.840)	0.810 (0.729-0.891)	0.944 (0.922-0.966)	0.947 (0.905-0.989)
Sensitivity (%)	81.0	82.4	92.5	94.1
Specificity (%)	65.5	73.3	85.7	84.4
Accuracy (%)	75.0	78.9	89.9	90.4
PPV/NPV (%)	78.9/68.3	83.1/72.3	91.2/87.8	90.6/90.0
PLR/NLR	2.35/0.29	3.09/0.24	6.48/0.09	6.05/0.07
PHC vs. NPHC (n/n)	268/703	85/173	280/699	73/177
AUROC(95%CI)	0.835 (0.806-0.863)	0.831 (0.780-0.863)	0.933 (0.916-0.951)	0.909 (0.868-0.950)
Sensitivity (%)	78.7	77.6	86.1	90.4
Specificity (%)	73.4	73.4	85.8	82.5
Accuracy (%)	74.9	74.8	85.9	84.8
PPV/NPV (%)	53.0/90.1	58.9/87.0	70.9/93.9	68.0/95.4
PLR/NLR	2.96/0.29	2.92/0.30	6.08/0.16	5.16/0.12

Note: F-M: the model established with fluorescence indicators; FAHB-M: the model established with fluorescence indicators, alpha-fetoprotein, hepatic function tests and blood cell analyses. PHC: primary hepatic carcinoma; NC: normal control; LC: liver cirrhosis; CH: chronic hepatitis; NPHC: non primary hepatic carcinoma (NC+LC+CH); AUROC: area under the receiver operating characteristic curve; CI: confidence interval; PPV/NPV: positive/negative predictive value; PLR/NLR: positive/negative likelihood ratio.

**Figure 4:**
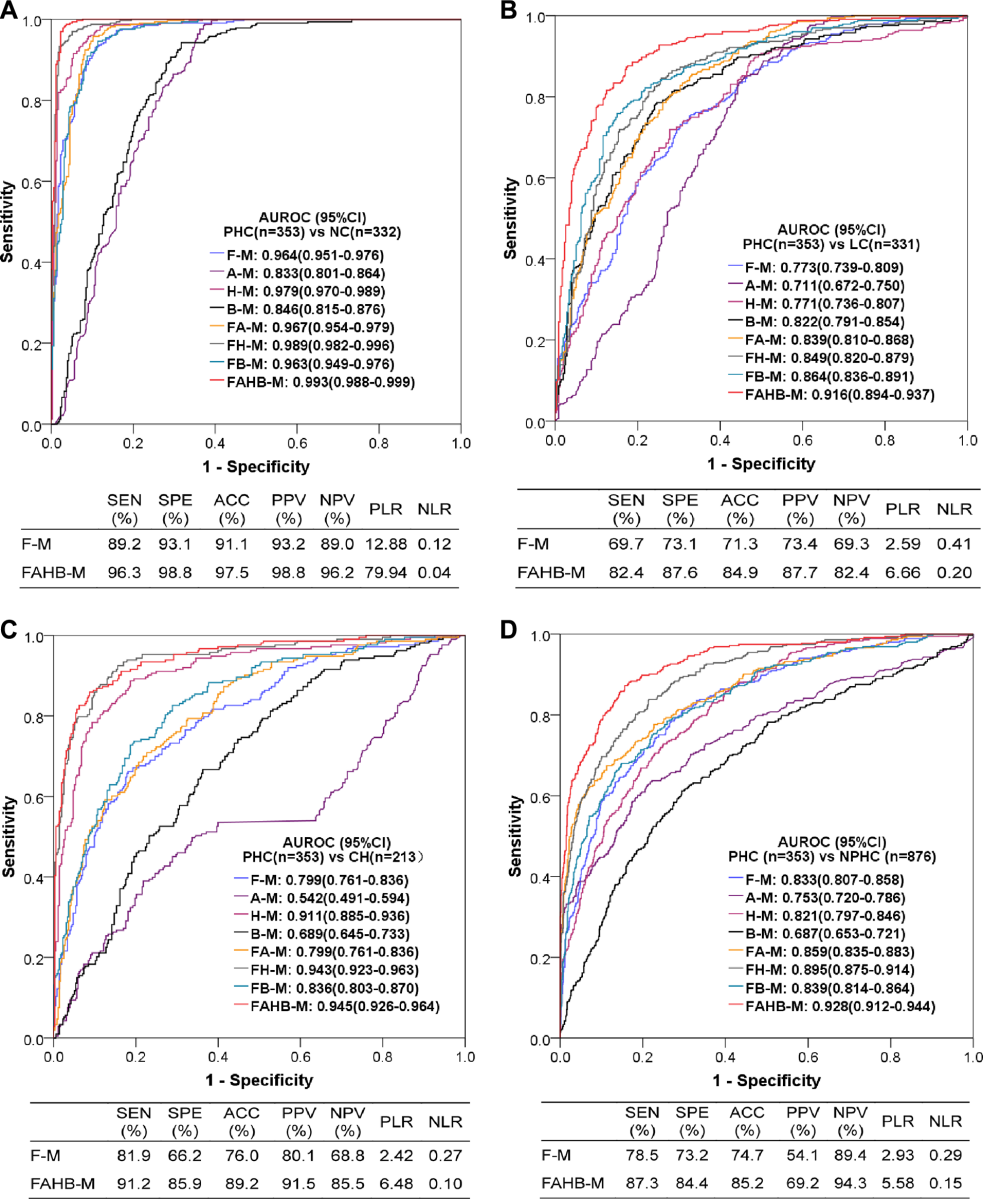
Diagnostic value of the models for diagnosing PHC vs. NC (**A**), LC (**B**), CH (**C**) and NPHC (**D**). AUROC: area under the receiver operating characteristic curve; CI: confidence interval; PHC: primary hepatic carcinoma; NC: normal control; LC: liver cirrhosis; CH: chronic hepatitis; NPHC: non-primary hepatic carcinoma (NC+LC+CH). Each model name is a combination of abbreviations representing fluorescence intensity (F), alpha-fetoprotein (A), hepatic function tests (H) and/or blood cell analyses (B) with the model (−M), which indicates the covariates used during modeling; for example, FAHB-M was established with indicators of fluorescence intensity, alpha-fetoprotein, hepatic function tests and blood cell analyses. SEN: sensitivity; SPE: specificity; ACC: accuracy; PPV: positive predictive value; NPV: negative predictive value; PLR: positive likelihood ratio; NLR: negative likelihood ratio.

Because the AUROCs of neither model F-M nor FAHB-M were significantly differed between the training and validation sets (all 95% CIs overlapped between two sets) and the accuracies of both models were similar between the two data sets, all subjects were used to evaluate the diagnostic value of these models. The ROC curves and their AUROCs of all models and the diagnostic performances of models F-M and FAHB-M are shown in Figure 4. In addition to models F-M and FAHB-M, model H-M was also excellent in discriminating PHC from NC and CH; model B-M was good for discriminating PHC from LC; furthermore, each blood laboratory test combined with serum fluorescence to establish models improved the diagnostic value for PHC.

### Diagnostic value of the models for PHC subgroups based on serum AFP levels

**Figure 5:**
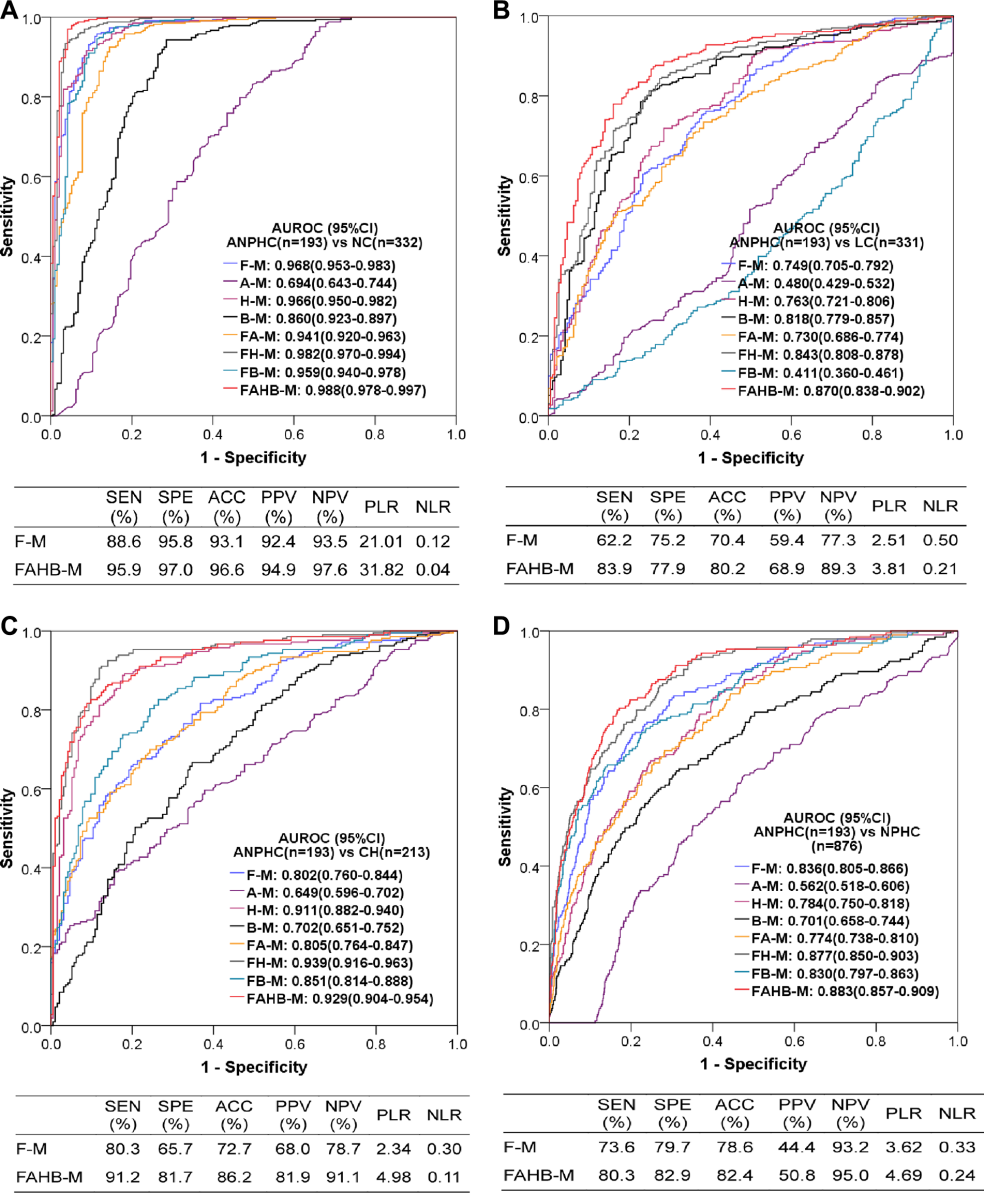
Diagnostic value of the models for diagnosing AFP-negative PHC vs. NC (**A**), LC (**B**), CH (**C**) and NPHC (**D**). AUROC: area under the receiver operating characteristic (ROC) curve; CI: confidence interval; ANPHC: AFP-negative primary hepatic carcinoma; NC: normal control; LC: liver cirrhosis; CH: chronic hepatitis; NPHC: non-primary hepatic carcinoma (NC+LC+CH). Each model name is a combination of abbreviations indicating fluorescence intensity (F), alpha-fetoprotein (A), hepatic function testresults (H) and blood cell analyses (B) with the model (−M), which indicate the covariates used during modeling; for example, FAHB-M was established with indicators of fluorescence intensity, alpha-fetoprotein, hepatic function test results and blood cell analyses. SEN: sensitivity; SPE: specificity; ACC: accuracy; PPV: positive predictive value; NPV: negative predictive value; PLR: positive likelihood ratio; NLR: negative likelihood ratio.

Of the 353 PHC patients, 193 patients exhibited a serum AFP < 20 ng/mL, 40 patients exhibited an AFP 20–<200 ng/mL, and 120 patients exhibited an AFP ≥200 ng/mL. The ROC curves and AUROCs of all models as well as the diagnostic performances of models F-M and FAHB-M for the AFP-negative PHC are shown in Figure 5. The F-M model was valuable for discriminating AFP-negative PHC from NC, LC, CH and NPHC, with excellent, fair, good and good AUROCs, respectively, and the FAHB-M model robustly discriminated AFP-negative PHC from NC, LC, CH and NPHC, with excellent, good, excellent and good AUROCs, respectively. The model H-M also effectively diagnosed AFP-negative PHC, especially combined with serum fluorescence.

The diagnostic value of model F-M for PHC was independent of the serum AFP levels, with similar AUROCs and diagnostic performances between AFP levels above and below 200 ng/mL. However, the diagnostic value of the FAHB-M model for PHC related to the AFP level, with better performances for diagnosing AFP-positive than AFP-negative PHCs. For more details refer to Table S4.

### Diagnostic value of the models for PHC subgroups based on BCLC stages

**Figure 6:**
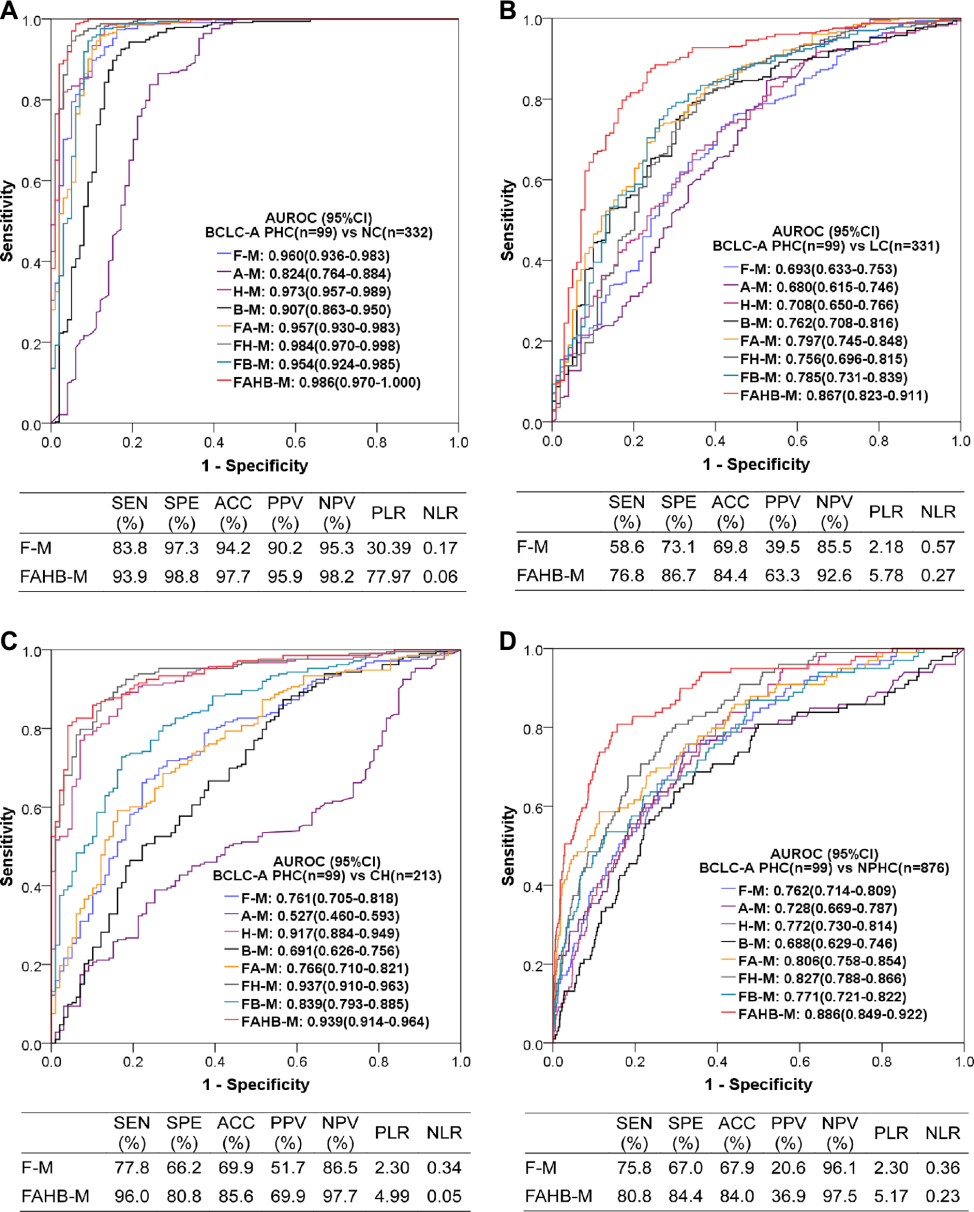
Diagnostic value of the models for diagnosing PHC at BCLC stage A vs. NC (**A**), LC (**B**), CH (**C**) and NPHC (**D**). AUROC: area under the receiver operating characteristic (ROC) curve; CI: confidence interval; BCLC-A: Barcelona Clinic Liver Cancer stage A; PHC: primary hepatic carcinoma; NC: normal control; LC: liver cirrhosis; CH: chronic hepatitis; NPHC: non-primary hepatic carcinoma (NC+LC+CH). Each model name is a combination of abbreviations representing fluorescence intensity(F), alpha-fetoprotein (A), hepatic function test results (H) and/or blood cell analyses (B) with the model (−M), which indicate the covariates used during modeling; for example, FAHB-M was established with indicators of fluorescence intensity, alpha-fetoprotein, hepatic function test results and blood cell analyses. SEN: sensitivity; SPE: specificity; ACC: accuracy; PPV: positive predictive value; NPV: negative predictive value; PLR: positive likelihood ratio; NLR: negative likelihood ratio.

Of the 353 PHC patients, the BCLC stage A, B, C, and D patients were 99, 170, 56, and 28, respectively. The ROC curves and AUROCs of all models and the diagnostic performances of models F-M and FAHB-M to discriminate BCLC stage A from NC, LC, CH and NPHC are shown in Figure 6. Model F-M was valuable for discriminating PHC at BCLC stage A from NC, LC, CH and NPHC, with excellent, poor, fair and fair AUROCs, respectively, and model FAHB-M was robust for discriminating PHC at BCLC stage A from NC, LC, CH and NPHC, with excellent, good, excellent and good AUROCs, respectively. Model H-M is also suitable for discriminating BCLC stage A PHC from NC and CH, with excellent AUROCs. More details about the diagnostic performances of models F-M and FAHB-M are shown in Table S5.

### Diagnostic value of the models for PHC subgroups based on tumor sizes

**Figure 7:**
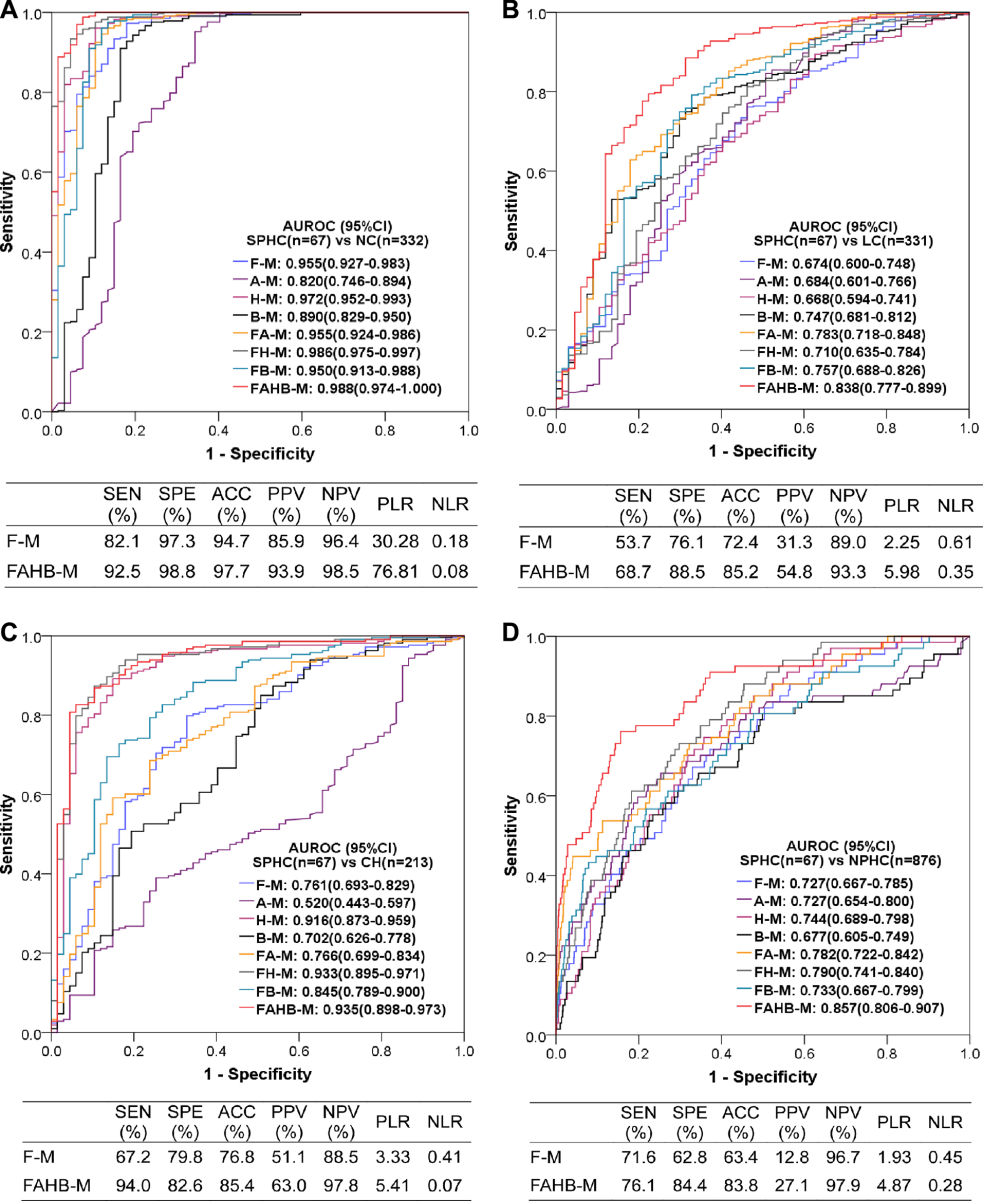
Diagnostic value of the models for discriminating small PHC from NC (**A**), LC (**B**), CH (**C**) and NPHC (**D**). AUROC: area under the receiver operating characteristic (ROC) curve; CI: confidence interval; SPHC: small primary hepatic carcinoma; NC: normal control; LC: liver cirrhosis; CH: chronic hepatitis; NPHC: non-primary hepatic carcinoma (NC+LC+CH). Each model name is a combination of abbreviations representing fluorescence intensity (F), alpha-fetoprotein (A), hepatic function tests (H) and/or blood cell analyses (B) with the model (−M), which indicate the covariates used during modeling; for example, FAHB-M was established with indicators of fluorescence intensity, alpha-fetoprotein, hepatic function tests and blood cell analyses. SEN: sensitivity; SPE: specificity; ACC: accuracy; PPV: positive predictive value; NPV: negative predictive value; PLR: positive likelihood ratio; NLR: negative likelihood ratio.

The tumor sizes were known in 292 of 353 PHC patients, specifically, 67 patients with tumors ≤ 3 cm (small PHC), 109 patients with tumors ≤ 5 cm and 183 patients with tumors > 5 cm. The ROC curves and AUROCs of all models as well as the diagnostic performances of both the F-M and FAHB-M models for small PHC vs. NC, LC, CH and NPHC are shown in Figure 7. Model F-M was valuable for discriminating small PHC from NC, LC, CH and NPHC, with excellent, poor, poor and fair AUROCs, respectively, and model FAHB-M was robust for discriminating small PHC from NC, LC, CH and NPHC, with excellent, good, excellent and good AUROCs, respectively. Model H-M was also suitable for discriminating small PHC from NC and CH, with excellent AUROCs. The detailed diagnostic performances are shown in Table S6.

### Comparison of the diagnostic values of models F-M and FAHB-M for PHC diagnosed by pathology and imaging

Of the 353 PHC patients, 96 were diagnosed based on histology, and the others were diagnosed based on imaging. We compared the diagnostic values of models F-M and FAHB-M for PHC with or without pathology and found that the AUROCs did not significantly differ (all 95%CIs overlapped) between the PHCs diagnosed by pathology and imaging (Table S7). This finding indicates that the diagnosis of PHC based on imaging is as reliable as that based on pathology, and both models are valuable in clinical practice.

### Comparison of the positive rates of models F-M and FAHB-M between HCC and ICC

Of the 96 PHC patients with histological results, 69 were HCC and 27 were ICC. We calculated the positive rates of the F-M and FAHB-M models as well as that of AFP in patients of HCC, ICC and unknown histology. Both models F-M and FAHB-M had significantly higher positive rates than AFP for diagnosing HCC, ICC and tumors of unknown histology. For model FAHB-M, the positive rate was significantly higher for HCC than for ICC, but model F-M did not significantly differ between HCC and ICC. For HCC, model FAHB-M exhibited a significantly higher positive rate than model F-M (*P* = 0.036), and model F-M exhibited a higher positive rate for ICC than model FAHB-M (*P* = 0.202). For more details, refer to Table S8.

## DISCUSSION

In this study, we developed a novel method by which to simultaneously measure the serum autofluorescence and cfDNA-related fluorescence using a real-time PCR system as a convenient fluorometer with temperature and time control “functions”. Compared with the conventional method for detecting serum autofluorescence, which relies on the use of a spectrofluorometer [10–12], our method is rapid, high-throughput and efficient. All operations could be completed within half an hour. A single assay provided 8 original and 12 derived indicators of serum fluorescence. Specifically, the serum cfDNA-related fluorescence can be concurrently measured in the same tube, which minimizes sampling errors and provides the most reliable self-comparisons.

Some single serum fluorescence indicators were excellent for differentiating PHC from NC (maximum AUROC 0.93), in accordance with a previous report [18], but only a few indicators were fair for differentiating PHC from LC, CH and NPHC (maximum AUROCs 0.70–0.73). However, indicators usually differed in their diagnostic value. Therefore, we combined multiple fluorescence indicators and developed the diagnostic model F-M, which was valuable for diagnosing PHC, including early, small and AFP-negative PHCs; the diagnostic value of this model was better than or equal to that of AFP.

The analyses of demographic and laboratory test results showed that hepatic function tests and blood cell analyses were significantly different among the PHC, LC, CH and NC groups. Therefore, we established diagnostic models H-M (based on hepatic function tests) and B-M (based on blood cell analyses), which performed better than AFP for differentiating PHC from benign liver diseases and corroborates the previous report that liver function tests were valuable for discriminating HCC from liver fibrosis [19]. Furthermore, we combined fluorescence parameters with hepatic function tests and blood cell analyses to build the FH-M and FB-M diagnostic models and obtained higher AUROCs, as expected. Finally, we combined serum fluorescence with AFP, hepatic function tests and blood cell analyses to establish a “full” diagnostic model FAHB-M, which showed robust performances for discriminating not only all PHC but also BCLC stage A, small and AFP-negative PHCs from NC, LC, CH and NPHC. Overall, the performance of this model was better than that of the reported biomarkers AFP, lens culinaris agglutinin-reactive fraction of AFP (AFP-L3), DCP, GPC3, osteopontin and GP73 [20].

Of the 96 PHC cases confirmed by histology, 69 cases were HCC and 27 cases were ICC. We compared the diagnostic performances of models F-M and FAHB-M between HCC and ICC and found that model F-M was highly sensitive for ICC detection (85.2%) and model FAHB-M was very sensitive for HCC detection (94.2%), suggesting that the combination of both models may be ideal to detect PHC. Additionally, model F-M highly sensitive to ICC suggests that ICC serum contains unique components that warrant study.

At the wavelengths examined in the present study (excitation 490 nm, emission 525 nm), autofluorescence can be given by a miscellaneous of endogenous fluorophores, including proteins, lipids, retinoids, lipofuscins, lipofuscin-like lipopigments, ceroids [21], and bilirubin [22]. Although porphyrins are important endogenous fluorophores and elevated in liver diseases [23], they barely contribute the autofluorescence in the present study due to their fluorescence excitation/emission conditions far from those here investigated [21]. These endogenous fluorescent substances vary by metabolic condition. Liver diseases may cause variations in the extracellular matrix, collagen enzymes, cell factors and porphyrin derivatives in the blood, which consequently alter the serum/plasma fluorescence intensity and lead to blood autofluorescence intensity that differs from those observed under healthy conditions. The serum autofluorescence intensity reportedly gradually increased as liver fibrosis progressed in rats [24]. Changes in the serum autofluorescence have been exploited for the diagnosis of chronic liver diseases [10–12]. Additionally, It was reported that indirect bilirubin was more fluorescent than direct bilirubin in bile [25], but we did not found this phenomenon in serum as the correlation coefficients of indirect bilirubin were similar with direct bilirubin (Table S2).

To our knowledge, the present study is the first to combine serum autofluorescence and cfDNA-related fluorescence for diagnostic applications. Although circulating cfDNA is usually detected by quantitative DNA amplification, it can be directly, simply and accurately detected using the fluorescent dyes PicoGreen [26] or SYBR Gold [27], and these measurements correlate well with the results of quantitative methods. In the present study, the serum cfDNA-related fluorescence intensity was obtained using EvaGreen, a fluorescent dye that is extremely sensitive to double-stranded DNA in real-time PCR assays [28]. All four fluorescence indicators derived from the difference between the presence and absence of EvaGreen significantly differed between the PHC and LC, CH and NC groups, despite the fact that the related original indicators may be insignificant.

A recent meta-analysis showed that cfDNA levels detected quantitatively are more valuable than serum AFP levels for diagnosing HCC, however, the independent use of the cfDNA test for HCC diagnosis is not recommended because this test is not robust for diagnosis [15]. In the present study, the diagnostic performances of single fluorescence indicators were not ideal, but the combination of multiple fluorescence indicators markedly improved the diagnostic value, and moreover, combining fluorescence indicators with routine laboratory tests further improved the diagnostic value. More importantly, combining hepatic function tests makes the diagnostic models more “PHC-specific”, which theoretically helps to avoid the “tumor-specific” shortage of cfDNA. Therefore, combining available laboratory data with specific tests for a precise diagnosis is a meaningful issue that warrants further study.

Although the models that combined serum fluorescence with routine laboratory tests showed robust diagnostic performance, their diagnostic power can be improved because some “bias” exists in the patient cohorts. The role of AFP was likely weakened in the models. To address the diagnosis of AFP-negative PHC, we collected as many AFP-negative patients as possible. These patients made up 54.7% (193/353) of the PHC group, a much higher proportion than that in a clinical setting (30–40%). Conversely, patients in the CH group were hospitalized with relatively severe disease; therefore, the proportion of patients (31.0%) with AFP levels > 20 ng/mL was larger than that in clinical practice [29].

In conclusion, the present study seamlessly integrates convenient analysis and powerful performance for diagnosing PHC, in accordance with the concept of “sample-to-answer solutions” in current diagnostics [30]. We developed a novel, simple and high-throughput method to rapidly and simultaneously measure serum autofluorescence and cfDNA-related fluorescence using a conventional real-time PCR system; this method was used to measure both types of serum fluorescence in 1229 specimens. The serum fluorescence parameters differed between PHC and LC, CH and NC groups to various extents and were not associated with age, gender or AFP levels. The diagnostic models established with the serum fluorescence parameters, particularly combined with AFP, hepatic function tests and blood cell analyses, showed robust diagnostic performances for PHC (including ICC), especially for AFP-negative, BCLC stage A and small PHCs. To our knowledge, this study is the first to systematically and concurrently evaluate the diagnostic significance of serum autofluorescence and cfDNA-related fluorescence alone and in combination with simple laboratory tests for PHC, especially for early detection, based on a large sample including LC, CH and NC groups.

## MATERIALS AND METHODS

### Collection of serum specimens and clinical data

Leftover serum specimens (initially drawn for routine laboratory tests) obtained prior to therapy were collected from hospitalized patients with liver diseases (PHC, LC and CH) and healthy subjects during routine check-ups at the First Affiliated Hospital of Nanchang University during 2013–2014. The samples were frozen at −80°C. Available clinical data were collected from these patients, including age, gender, blood biochemistry, serum tumor markers, medical imaging and pathology. PHC was diagnosed based on histology or other non-invasive diagnostic criteria (coincidental results of B type ultrasound and CT and/or MRI with or without elevated serum AFP) [31]. Early PHC refers to BCLC stage A. Tumor ≤3 cm in size was classified as small PHC. AFP-negative PHC refers to a serum AFP level < 20 ng/mL. Cirrhosis and chronic hepatitis were diagnosed based on clinical manifestation, laboratory tests and liver medical imaging. Subjects exhibiting normal laboratory blood test results (hepatic function, kidney function, serum markers of hepatitis B virus and blood cell analyses), tumor marker test results, B-type ultrasound images of the upper abdomen, electrocardiogram and chest X rays during their check-up were classified as normal controls. This study was approved by the First Affiliated Hospital of Nanchang University Committee for Clinical Investigation, which determined that patient consent was not necessary.

### Measurement of serum fluorescence intensity

The StepOne Plus^TM^ Real-Time PCR system (Applied Biosystem, USA) was used to measure the serum FI. The excitation and emission wavelengths were 490 nm and 525 nm, respectively. These wavelengths are suitable for EvaGreen (Biotium, USA), which is a double-stranded nucleic acid dye used to detect products of real-time PCR amplification. During the measurement of serum FI, the reaction mixture was maintained at a certain temperature for 1 minute followed by data collection for 30 seconds.

### Optimization of conditions for the serum fluorescence intensity measurement of primary hepatic carcinoma

### Determination of the optimum serum volume

Pooled sera from PHC, LC, CH and NC subjects were separately prepared by mixing 20 random serum specimens of the same volume (50 μL). PCR tube strips (BIOplastics, the Netherlands) were filled with 0, 3, 6, 9, 12, or 15 μL of pooled serum, and each aliquot was diluted with ultrapure water to a total volume of 20 μL. The FI of each tube was detected at 37°C. The serum volume(s) with the largest (when > 1) or smallest (when < 1) FI ratio(s) of PHC to LC, CH and NC was (were) optimal.

### Determination of the optimum temperature

The optimum volume of pooled serum determined above was diluted with ultrapure water to 20 μL followed by FI measurement at several temperatures: 8, 12, 15, 20, 25, 30, or 37°C. The temperature(s) with the largest (when > 1) or smallest (when < 1) FI ratio(s) of PHC to LC, CH and NC was (were) optimal.

### Determination of the optimum EvaGreen volume

The optimum volume of pooled serum was diluted with ultrapure water to 20 μL and then mixed with 1, 2, 3, or 4 μL of 2× EvaGreen (all diluted with ultrapure water to 25 μL). The FI of each tube was then detected at the optimum temperature determined above. The EvaGreen point(s) with the largest (when > 1) or smallest (when < 1) FI ratio(s) of PHC to LC, CH and NC was (were) optimal.

### Determination of the optimum cycles

The optimum pooled serum volumes with or without the optimum volume of 2× EvaGreen were diluted with ultrapure water up to 20 μL, and their FIs were detected at the optimum temperature from cycle 1 to 10. The cycle(s) with the largest (when > 1) or smallest (when < 1) FI ratio(s) of PHC to LC, CH and NC was (were) optimal.

### Fluorescence intensity measurement of serum specimens

**Figure 8:**
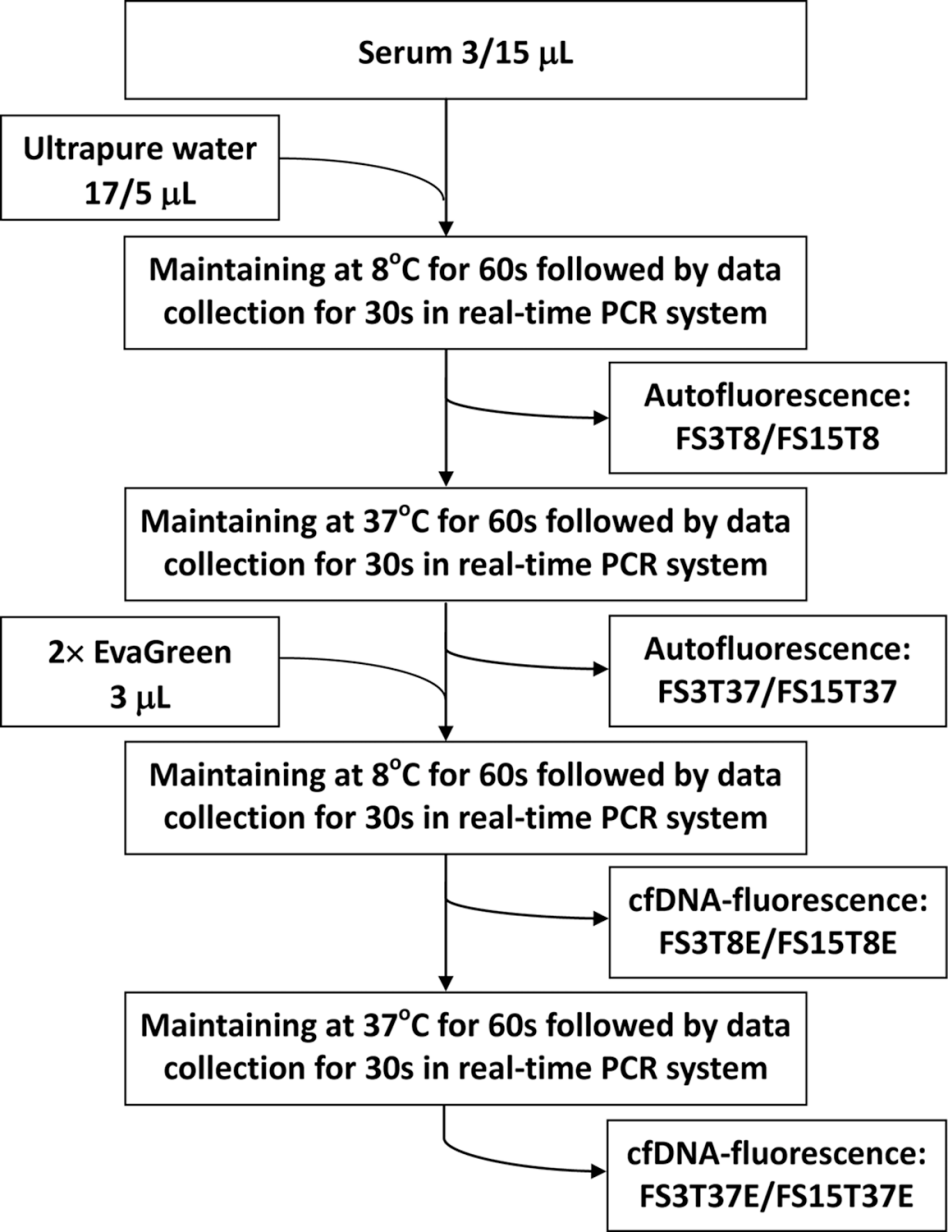
Diagram of the measurement of serum autofluorescence and cell-free DNA-related fluorescence FS3T8, FS15T8, FS3T37, FS15T37, FS3T8E, FS15T8E, FS3T37E and FS15T37E: the names of 8 original fluorescence indicators. Each indicator name is an abbreviation indicating the fluorescence intensity (F) of serum (S) 3 μL (3) or 15 μL (15) at a given temperature (T) 8°C (8) or 37°C (37) in the presence (E) or absence of EvaGreen.

Serum specimens stored at −80°C were thawed at room temperature. The FIs of each serum specimen were measured at the optimum conditions determined above. Figure 8 shows the procedure used to measure the serum autofluorescence and cfDNA-related fluorescence as well as the 8 original fluorescence indicators obtained (FS3T8, FS3T37, FS3T8E, FS3T37E, FS15T8, FS15T37, FS15T8E, and FS15T37E). Twelve difference indicators were calculated by subtracting one of the 8 original indicators from another related indicator (FSDT8, FSDT37, FSDT8E, FSDT37E, FTDS3, FTDS3E, FTDS15, FTDS15E, FEDS3T8, FEDS3T37, FEDS15T8, and FEDS15T37). These FI indicators were named based on a combination of several abbreviations: F (fluorescence), S (serum), T (temperature), E (EvaGreen), SD (FI difference between 3 μL and 15 μL of serum), TD (FI difference between 8°C and 37°C) and/or ED (FI difference in the presence and absence of EvaGreen) followed by related parameters of 3 (3 μL), 15 (15 μL), 8 (8°C) or 37 (37°C). This name indicated the serum fluorescence intensity measured under a specific condition; for example, FEDS15T37 indicates the fluorescence intensity difference for 15 μL of serum in the presence and absence of EvaGreen at 37°C.

### Statistical analyses and diagnostic significance evaluation

All statistical analyses were performed using SPSS Statistics 20.0 (IBM, USA). Continuous variables are expressed as the mean and standard deviation (mean ± SD) and compared between groups using a one-way ANOVA. Categorical variables are expressed as a frequency or percentage and compared using the Pearson's chi-squared test or Fisher's exact test. Correlations among variables were analyzed with Pearson correlation analysis. All statistical tests were two-sided, and *P* < 0.05 was considered to indicate significant differences.

A multivariate multinomial logistic stepwise regression analysis was used to establish diagnostic models for differentiating PHC from NC, LC, CH and NPHC. The “estimated response probabilities” of models were saved during logistic regressions and used as the test variables in ROC curve analyses.

The diagnostic significance of a variable or model for PHC vs. NC, LC, CH or NPHC was evaluated based on the AUROC according to the following criteria: 0.90–1.00, excellent; 0.80–0.89, good; 0.70–0.79, fair; 0.60–0.69, poor, and 0.50–0.59, fail. The point with the largest Youden's index in the “coordinate points of ROC curve” was selected as the cut-off value to calculate the diagnostic performance (sensitivity, specificity, accuracy, positive/negative predictive value, and positive/negative likelihood ratio).

## SUPPLEMENTARY MATERIALS TABLES



















## Abbreviations

AFP: alpha-fetoprotein
ALT: alanine aminotransferase
AST: aspartate aminotransaminase
AUROC: area under the receiver operating characteristic curve
cfDNA: circulating cell-free DNA
CH: chronic hepatitis
CI: confidence interval
DBIL: direct serum bilirubin
FI: fluorescence intensity
GLB: serum gamma-globins
HCC: hepatocellular carcinoma
IBIL: indirect serum bilirubin
ICC: intrahepatic cholangiocarcinoma
LC: liver cirrhosis
NC: normal control
NPHC: total of normal control liver cirrhosis and chronic hepatitis
OR: odds ratio
ORa: adjusted odds ratio for age and gender
PHC: primary hepatic carcinoma
PLT: platelet
Ra: correlation coefficients of a fluorescence intensity indicator with age
Rg: correlation coefficients of a fluorescence intensity indicator with gender
RBC: red blood cell
ROC: the receiver operating characteristic curve
TBIL: total serum bilirubin
TP: total serum protein
WBC: white blood cell
