# Supplementary material for: Simple and robust diagnosis of early, small and AFP-negative primary hepatic carcinomas: an integrative approach of serum fluorescence and conventional blood tests
Source: Oncotarget. 2016 Aug 31;7(39):64053–70. doi: 10.18632/oncotarget.11771 (PMC5325425; doi:10.18632/oncotarget.11771)
Supplement: Supplementary file 1 [file oncotarget-07-64053-s001.pdf]

Simple and robust diagnosis of early, small and AFP-negative primary hepatic carcinomas: an integrative approach of serum fluorescence and conventional blood tests

Supplementary Materials

Supplementary Table S1: Associations of serum fluorescence indicators with age and gender. See Supplementary_Table_S1

Supplementary Table S2: Correlations of serum fluorescence intensity with blood laboratory test results. See Supplementary_Table_S2

Supplementary Table S3: AUROCs of single indicators of serum fluorescence intensity and laboratory blood tests for diagnosing PHC. See Supplementary_Table_S3

Supplementary Table S4: Diagnostic value of models F-M and FAHB-M for PHC subgroups based on serum AFP levels. See Supplementary_Table_S4

Supplementary Table S5: Diagnostic value of models F-M and FAHB-M for PHC subgroups based on BCLC stages. See Supplementary_Table_S5

Supplementary Table S6: Diagnostic value of models F-M and FAHB-M for PHC subgroups based on tumor sizes. See Supplementary_Table_S6

Supplementary Table S7: Comparison of the diagnostic values of models F-M and FAHB-M for PHC diagnosed by pathology and imaging. See Supplementary_Table_S7

Supplementary Table S8: Positive rates of models F-M and FAHB-M and AFP in PHC with different histological types. See Supplementary_Table_S8
