# Supplementary material for: Simple and robust diagnosis of early, small and AFP-negative primary hepatic carcinomas: an integrative approach of serum fluorescence and conventional blood tests
Source: Oncotarget. 2016 Aug 31;7(39):64053–70. doi: 10.18632/oncotarget.11771 (PMC5325425; doi:10.18632/oncotarget.11771)
Supplement: Supplementary file 2 [file oncotarget-07-64053-s002.docx]

**Table S1 Associations of serum fluorescence indicators with age and gender**

|  | OR (%95 CI) | ORa (%95 CI) | Ra | Rg |
| --- | --- | --- | --- | --- |
| PHC(n=353) vs. LC(n=331) | |  |  |  |
| FS3T37E | 2.315(1.358-3.946) | 2.190 (1.278-3.754) | -0.111 | 0.024 |
| FS3T8E | 2.167(1.338-3.511) | 2.078(1.277-3.382) | -0.091 | 0.026 |
| FS15T8E# | 1.536(1.011-2.332) | 1.452(0.953-2.214) | -0.113 | -0.010 |
| PHC(n=353) vs. CH(n=213) | |  |  |  |
| FS3T8 | 0.480(0.338-0.681) | 0.223(0.141-0.353) | -0.405 | -0.091 |
| FS3T37 | 0.612(0.405-0.925) | 0.262(0.155-0.444) | -0.357 | -0.083 |
| FS15T37# | 1.761(1.207-2.569) | 1.174(0.750-1.839) | -0.121 | -0.098 |
| FS15T8E# | 2.124(1.294-3.488) | 1.243(0.688-2.244) | -0.123 | -0.114 |
| FS15T37E | 2.916(1.826-4.658) | 1.989(1.152-3.434) | -0.053 | -0.101 |
| PHC(n=353) vs. NC(n=332) | |  |  |  |
| FS3T8 | 62.512(28.177-138.682) | 47.724(21.055-108.174) | 0.090 | -0.106 |
| FS3T37 | 155.507(61.299-394.500) | 113.163(43.657-293.325) | 0.079 | -0.102 |
| FS3T8E | 94.087(36.862-240.151) | 63.429(24.560-163.816) | 0.057 | -0.093 |
| FS3T37E | 357.886(117.192-1092.925) | 222.242(71.717-689.942) | 0.023 | -0.074 |
| FS15T8 | 630.485(232.439-1710.175) | 472.834(173.362-1289.627) | -0.007 | -0.143 |
| FS15T37 | 1006.031(350.112-2890.786) | 759.106(262.750-2193.116) | -0.076 | -0.129 |
| FS15T8E | 4181.275(1157.171-15108.449) | 2972.534(811.433-10889.327) | 0.029 | -0.146 |
| FS15T37E | 5613.932(1504.663-20945.703) | 3948.553(1050.949-14835.226) | -0.048 | -0.122 |
| PHC(n=353) vs. NPHC(n=876) | |  |  |  |
| FS3T8 | 1.510(1.156-1.972) | 1.352(1.024-1.786) | -0.009 | -0.095 |
| FS3T37 | 2.047(1.498-2.799) | 1.766(1.276-2.445) | -0.023 | -0.086 |
| FS3T8E | 2.840(1.916-4.209) | 2.479(1.648-3.728) | -0.001 | -0.060 |
| FS3T37E | 4.770(3.026-7.519) | 3.882(2.425-6.216) | -0.029 | -0.048 |
| FS15T8 | 2.916(2.233-3.807) | 2.536(1.926-3.339) | -0.021 | -0.096 |
| FS15T37 | 3.086(2.388-3.988) | 2.686(2.063-3.497) | -0.031 | -0.091 |
| FS15T8E | 5.211(3.663-7.414) | 4.439(3.091-6.374) | -0.004 | -0.087 |
| FS15T37E | 4.538(3.306-6.229) | 3.846(2.780-5.321) | -0.030 | -0.082 |
| Note: Only the indicators with a significant OR are shown here, and ORs that became insignificant after adjustment are labeled with “#”. Because the magnitude of original FI indicators was too large to appropriately show the ORs, all indicators were transformed with a natural logarithm prior to the calculation. ORa: the adjusted odds ratio for gender and age. Ra and Rg: correlation coefficients of a fluorescence intensity indicator with age and gender from the “Correlation Matrix” given by a binary logistic regression in the ORa calculation. PHC: primary hepatic carcinoma; LC: liver cirrhosis; CH: chronic hepatitis; NC: normal control. The fluorescence indicator names are the combinations of several abbreviations representing the fluorescence intensity (F) of 3μL (S3) or 15μL (S15) of serum at a detection temperature of 8°C (T8) or 37°C (T37) in the presence (E) or absence of EvaGreen. | | | | |
