# Supplementary material for: Simple and robust diagnosis of early, small and AFP-negative primary hepatic carcinomas: an integrative approach of serum fluorescence and conventional blood tests
Source: Oncotarget. 2016 Aug 31;7(39):64053–70. doi: 10.18632/oncotarget.11771 (PMC5325425; doi:10.18632/oncotarget.11771)
Supplement: Supplementary file 3 [file oncotarget-07-64053-s003.docx]

**Table S2 Correlations of serum fluorescence intensity with blood laboratory test results**

|  | Correlation coefficient | | | | | | | |
| --- | --- | --- | --- | --- | --- | --- | --- | --- |
|  | FS3T8 | FS3T37 | FS3T8E | FS3T37E | FS15T8 | FS15T37 | FS15T8E | FS15T37E |
| AFP | 0.063* | 0.081** | 0.101** | 0.118** | 0.102** | 0.094** | 0.126** | 0.107** |
| ALT | 0.251** | 0.202** | 0.243** | 0.180** | 0.125** | 0.083** | 0.114** | 0.072* |
| AST | **0.315**** | 0.258** | **0.323**** | 0.252** | 0.163** | 0.110** | 0.150** | 0.099** |
| TBIL | **0.530**** | **0.383**** | **0.514**** | **0.351**** | 0.133** | 0.033 | 0.080** | -0.008 |
| DBIL | **0.524**** | **0.374**** | **0.510**** | **0.344**** | 0.124** | 0.025 | 0.072* | -0.016 |
| IBIL | **0.511**** | **0.377**** | **0.492**** | **0.346**** | 0.144** | 0.049 | 0.092** | 0.008 |
| TP | -0.287** | -0.279** | -0.229** | -0.233** | -0.291** | -0.264** | -0.259** | -0.244** |
| ALB | **-0.415**** | **-0.415**** | **-0.356**** | **-0.370**** | **-0.429**** | **-0.389**** | **-0.388**** | **-0.363**** |
| GLP | 0.136** | 0.147** | 0.142** | 0.155** | 0.150** | 0.135** | 0.142** | 0.130** |
| WBC | 0.023 | -0.005 | 0.111** | 0.064* | -0.056 | -0.070* | -0.017 | -0.052 |
| RBC | -0.227** | -0.234** | -0.169** | -0.197** | -0.258** | -0.241** | -0.220** | -0.222** |
| Hb | -0.159** | -0.185** | -0.103** | -0.152** | -0.239** | -0.239** | -0.208** | -0.226** |
| PLT | -0.180** | -0.190** | -0.096** | -0.119** | -0.242** | -0.232** | -0.200** | -0.210** |
| Note: * P<0.05, ** P<0.01. The fluorescence indicator names are combinations of abbreviations representing the fluorescence intensity (F) of 3μL (S3) or 15μL (S15) serum samples at a detection temperature of 8°C (T8) or 37°C (T37) in the presence (E) or absence of EvaGreen. AFP: alpha-fetoprotein; ALT: alanine transaminase; AST: aspartate transaminase; TBIL: total serum bilirubin; DBIL: direct serum bilirubin; IBIL: indirect serum bilirubin; TP: total serum protein; ALB: serum albumin; GLB: serum gamma-globins; WBC: white blood cell; RBC: red blood cell; Hb: hemoglobin; PLT: platelet. | | | | | | | | |
