# Supplementary material for: Simple and robust diagnosis of early, small and AFP-negative primary hepatic carcinomas: an integrative approach of serum fluorescence and conventional blood tests
Source: Oncotarget. 2016 Aug 31;7(39):64053–70. doi: 10.18632/oncotarget.11771 (PMC5325425; doi:10.18632/oncotarget.11771)
Supplement: Supplementary file 4 [file oncotarget-07-64053-s004.docx]

**Table S3 AUROCs of single indicators of serum fluorescence intensity and laboratory blood tests for diagnosing PHC**

|  | AUROC | | | |
| --- | --- | --- | --- | --- |
|  | PHC(n=353) vs. NC(n=332) | PHC(n=353) vs. LC(n=331) | PHC(n=353) vs. CH(n=213) | PHC(n=353) vs. NPHC(n=876) |
| Fluorescence intensity |  |  |  |  |
| FS3T8 | 0.766** | 0.532 | 0.601** | 0.564** |
| FS3T37 | 0.789** | 0.522 | 0.570** | 0.584** |
| FS3T8E | 0.723** | 0.570** | 0.544 | 0.600** |
| FS3T37E | 0.759** | 0.562** | 0.504 | 0.621** |
| FS15T8 | 0.910** | 0.522 | 0.508 | 0.649** |
| FS15T37 | 0.925** | 0.518 | 0.568** | 0.671** |
| FS15T8E | 0.907** | 0.541 | 0.569** | 0.687** |
| FS15T37E | 0.920** | 0.523 | 0.614** | 0.696** |
| FSDT8 | 0.904** | 0.515 | 0.618** | 0.676** |
| FSDT37 | 0.910** | 0.518 | 0.624** | 0.679** |
| FSDT8E | 0.901** | 0.514 | 0.624** | 0.687** |
| FSDT37E | 0.903** | 0.501 | 0.629** | 0.684** |
| FTDS3 | 0.712** | 0.544* | 0.633** | 0.531 |
| FTDS3E | 0.652** | 0.579** | 0.585** | 0.567** |
| FTDS15 | 0.844** | 0.521 | 0.563* | 0.607** |
| FTDS15E | 0.828** | 0.573** | 0.524 | 0.646** |
| FEDS3T8 | 0.677** | 0.708** | 0.712** | 0.563** |
| FEDS3T37 | 0.671** | 0.690** | 0.727** | 0.562** |
| FEDS15T8 | 0.648** | 0.712** | 0.667** | 0.565** |
| FEDS15T37 | 0.664** | 0.682** | 0.674** | 0.549** |
| AFP | 0.833** | 0.765** | 0.610** | 0.753** |
| Hepatic function tests |  |  |  |  |
| ALT | 0.827** | 0.624** | 0.862** | 0.583** |
| AST | 0.905** | 0.612** | 0.695** | 0.649** |
| TBIL | 0.737** | 0.573** | 0.628** | 0.531 |
| DBIL | 0.792** | 0.591** | 0.625** | 0.546* |
| TP | 0.865** | 0.642** | 0.515 | 0.588** |
| ALB | 0.958** | 0.698** | 0.599** | 0.623** |
| GLB | 0.633** | 0.509 | 0.586** | 0.575** |
| Blood cell analyses |  |  |  |  |
| WBC | 0.574** | 0.724** | 0.565** | 0.572** |
| RBC | 0.781** | 0.699** | 0.661** | 0.570** |
| Hb | 0.775** | 0.759** | 0.673** | 0.548** |
| PLT | 0.759** | 0.751** | 0.555* | 0.517 |
| Note: *P<0.05, **P<0.01. AUROC: the area under the receiver operator characteristic curve; PHC: primary hepatic carcinoma; LC: liver cirrhosis; CH: chronic hepatitis; NC: normal control. The names of the fluorescence indicators are combinations of abbreviations representing the fluorescence intensity (F) of 3μL (S3) or 15μL (S15) serum at a detection temperature of 8°C (T8) or 37°C (T37) in the presence (E) or absence of EvaGreen, as well as the fluorescence intensity differences between 3μL and 15μL serum samples (SD), between temperatures of 8°C and 37°C (TD) or between the presence and absence of EvaGreen (ED). AFP: alpha-fetoprotein; ALT: alanine transaminase; AST: aspartate transaminase; TBIL: total serum bilirubin; DBIL: direct serum bilirubin; TP: total serum protein; ALB: serum albumin; GLB: serum gamma-globins; WBC: white blood cell; RBC: red blood cell; Hb: hemoglobin; PLT: platelet. | | | | |
