# Supplementary material for: Simple and robust diagnosis of early, small and AFP-negative primary hepatic carcinomas: an integrative approach of serum fluorescence and conventional blood tests
Source: Oncotarget. 2016 Aug 31;7(39):64053–70. doi: 10.18632/oncotarget.11771 (PMC5325425; doi:10.18632/oncotarget.11771)
Supplement: Supplementary file 5 [file oncotarget-07-64053-s005.docx]

**Table S4 Diagnostic value of models F-M and FAHB-M for PHC subgroups based on serum AFP levels**

| Versus group | F-M | | | | |  | FAHB-M | | |
| --- | --- | --- | --- | --- | --- | --- | --- | --- | --- |
|  | PHC with serum AFP levels (ng/mL) | | | | |  | PHC with serum AFP levels(ng/mL) | | |
|  | <20  (n=193) | | <200  (n=233) | | ≥200  (n=120) |  | <20  (n=193) | <200  (n=233) | ≥200  (n=120) |
| **NC (n=332)** | |  | |  | |  |  |  |  |
| AUROC(95%CI) | 0.968(0.953-0.983) | | 0.968(0.955-0.982) | | 0.955(0.931-0.978) |  | 0.988(0.978-0.997) | 0.990(0.982-0.998) | 1.000(1.000-1.000) |
| Sensitivity (%) | 88.6 | | 88.0 | | 87.5 |  | 95.9 | 95.7 | 100.0 |
| Specificity (%) | 95.8 | | 95.8 | | 93.1 |  | 97.0 | 97.9 | 100.0 |
| Accuracy (%) | 93.1 | | 92.6 | | 91.6 |  | 96.6 | 97.0 | 100.0 |
| PPV/NPV (%) | 92.4/93.5 | | 93.6/91.9 | | 82.0/95.4 |  | 94.9/97.6 | 97.0/97.0 | 100.0/100.0 |
| PLR/NLR | 21.01/0.12 | | 20.86/0.13 | | 12.63/0.13 |  | 31.82/0.04 | 45.39/0.04 | -/0.00 |
| **LC (n=331)** | |  | |  | |  |  |  |  |
| AUROC(95%CI) | 0.749(0.705-0.792) | | 0.753(0.712-0.793) | | 0.814(0.769-0.858) |  | 0.870(0.838-0.902) | 0.879(0.850-0.908) | 0.987(0.977-0.996) |
| Sensitivity (%) | 62.2 | | 63.9 | | 77.5 |  | 83.9 | 81.5 | 96.7 |
| Specificity (%) | 75.2 | | 75.2 | | 73.1 |  | 77.9 | 81.6 | 95.2 |
| Accuracy (%) | 70.4 | | 70.7 | | 74.3 |  | 80.2 | 81.6 | 95.6 |
| PPV/NPV (%) | 59.4/77.3 | | 63.5/75.6 | | 51.1/90.0 |  | 68.9/89.3 | 75.7/86.3 | 87.9/98.7 |
| PLR/NLR | 2.51/0.50 | | 2.58/0.48 | | 2.88/0.31 |  | 3.81/0.21 | 4.42/0.23 | 20.0/0.04 |
| **CH (n=213)** | |  | |  | |  |  |  |  |
| AUROC(95%CI) | 0.802(0.760-0.844) | | 0.790(0.749-0.832) | | 0.815(0.768-0.861) |  | 0.929(0.904-0.954) | 0.931(0.908-0.955) | 0.971(0.955-0.987) |
| Sensitivity (%) | 80.3 | | 78.5 | | 86.7 |  | 91.2 | 91.4 | 93.3 |
| Specificity (%) | 65.7 | | 67.1 | | 66.2 |  | 81.7 | 82.6 | 91.5 |
| Accuracy (%) | 72.7 | | 73.1 | | 73.6 |  | 86.2 | 87.2 | 92.2 |
| PPV/NPV (%) | 68.0/78.7 | | 72.3/74.1 | | 59.1/89.8 |  | 81.9/91.1 | 85.2/89.8 | 86.2/96.1 |
| PLR/NLR | 2.34/0.30 | | 2.39/0.32 | | 2.56/0.20 |  | 4.98/0.11 | 5.26/0.10 | 11.04/0.07 |
| **NPHC(n=876)** | |  | |  | |  |  |  |  |
| AUROC(95%CI) | 0.836(0.805-0.866) | | 0.827(0.798-0.856) | | 0.843(0.803-0.883) |  | 0.883(0.857-0.909) | 0.895(0.872-0.918) | 0.993(0.987-0.999) |
| Sensitivity (%) | 73.6 | | 74.7 | | 81.7 |  | 80.3 | 82.4 | 97.5 |
| Specificity (%) | 79.7 | | 76.7 | | 73.5 |  | 82.9 | 83.3 | 97.1 |
| Accuracy (%) | 78.6 | | 76.3 | | 74.5 |  | 82.4 | 83.1 | 97.2 |
| PPV/NPV (%) | 44.4/93.2 | | 46.0/919.9 | | 29.7/96.7 |  | 50.8/95.0 | 56.8/94.7 | 82.4/99.6 |
| PLR/NLR | 3.62/0.33 | | 3.21/0.33 | | 3.08/0.25 |  | 4.69/0.24 | 4.94/0.21 | 34.16/0.03 |

Note: F-M: the model established with the indicators of fluorescence intensity; FAHB-M: the model established with the indicators of fluorescence intensity, alpha-fetoprotein, hepatic function tests and blood cell analyses; PHC: primary hepatic carcinoma; NC: normal control; LC: liver cirrhosis; CH: chronic hepatitis; NPHC: non primary hepatic carcinoma (NC+LC+CH); AUROC: area under the receiver operating characteristic curve; CI: confidence interval; PPV/NPV: positive/negative predictive value; PLR/NLR: positive/negative likelihood ratio.
