# Supplementary material for: Simple and robust diagnosis of early, small and AFP-negative primary hepatic carcinomas: an integrative approach of serum fluorescence and conventional blood tests
Source: Oncotarget. 2016 Aug 31;7(39):64053–70. doi: 10.18632/oncotarget.11771 (PMC5325425; doi:10.18632/oncotarget.11771)
Supplement: Supplementary file 6 [file oncotarget-07-64053-s006.docx]

**Table S5 Diagnostic value of models F-M and FAHB-M for PHC subgroups based on BCLC stages**

| Versus group | F-M | | |  | FAHB-M | | |
| --- | --- | --- | --- | --- | --- | --- | --- |
|  | PHC at BCLC stage | | |  | PHC at BCLC stage | | |
|  | A (n=99) | B (n=170) | C/D (n=84) |  | A (n=99) | B (n=170) | C/D (n=84) |
| **NC(n=332)** |  | |  |  |  |  |  |
| AUROC(95%CI) | 0.960(0.936-0.983) | 0.959(0.941-0.987) | 0.977(0.961-0.993) |  | 0.986(0.970-1.000) | 0.994(0.988-1.000) | 1.000(0.999-1.000) |
| Sensitivity (%) | 83.8 | 87.6 | 92.9 |  | 93.9 | 97.0 | 98.8 |
| Specificity (%) | 97.3 | 93.7 | 95.2 |  | 98.8 | 98.2 | 100.0 |
| Accuracy (%) | 94.2 | 91.6 | 94.7 |  | 97.7 | 98.0 | 99.8 |
| PPV/NPV (%) | 90.2/95.3 | 87.6/93.7 | 83.0/98.1 |  | 95.9/98.2 | 96.5/98.8 | 100.0/99.7 |
| PLR/NLR | 30.39/0.17 | 18.86/0.13 | 19.27/0.08 |  | 77.97/0.06 | 54.03/0.02 | -/0.01 |
| **LC(n=331)** |  | |  |  |  |  |  |
| AUROC(95%CI) | 0.693(0.633-0.753) | 0.800(0.759-0.841) | 0.814(0.764-0.865) |  | 0.867(0.823-0.911) | 0.947(0.928-0.966) | 0.908(0.875-0.941) |
| Sensitivity (%) | 58.6 | 71.8 | 77.4 |  | 76.8 | 87.1 | 84.5 |
| Specificity (%) | 73.1 | 75.2 | 73.1 |  | 86.7 | 90.6 | 81.9 |
| Accuracy (%) | 69.8 | 74.1 | 74.0 |  | 84.4 | 89.4 | 82.4 |
| PPV/NPV (%) | 39.5/85.5 | 59.8/83.8 | 42.2/92.7 |  | 63.3/92.6 | 82.7/93.2 | 54.2/95.4 |
| PLR/NLR | 2.18/0.57 | 2.90/0.38 | 2.88/0.31 |  | 5.78/0.27 | 9.30/0.14 | 4.66/0.19 |
| **CH(n=213)** |  | |  |  |  |  |  |
| AUROC(95%CI) | 0.761(0.705-0.818) | 0.823(0.782-0.864) | 0.793(0.736-0.850) |  | 0.939(0.914-0.964) | 0.943(0.922-0.965) | 0.954(0.925-0.985) |
| Sensitivity (%) | 77.8 | 87.1 | 67.9 |  | 96.0 | 89.4 | 96.4 |
| Specificity (%) | 66.2 | 65.7 | 76.5 |  | 80.8 | 85.9 | 86.9 |
| Accuracy (%) | 69.9 | 75.2 | 74.1 |  | 85.6 | 87.5 | 89.6 |
| PPV/NPV (%) | 51.7/86.5 | 67.0/86.4 | 53.3/85.8 |  | 69.9/97.7 | 83.5/91.0 | 74.3/98.4 |
| PLR/NLR | 2.30/0.34 | 2.54/0.20 | 2.89/0.42 |  | 4.99/0.05 | 6.35/0.12 | 7.34/0.04 |
| **NPHC(n=876)** |  | |  |  |  |  |  |
| AUROC(95%CI) | 0.762(0.714-0.809) | 0.849(0.816-0.882) | 0.882(0.845-0.919) |  | 0.886(0.849-0.922) | 0.947(0.930-0.965) | 0.940(0.914-0.966) |
| Sensitivity (%) | 75.8 | 76.5 | 79.8 |  | 80.8 | 85.3 | 90.5 |
| Specificity (%) | 67.0 | 79.7 | 82.1 |  | 84.4 | 90.4 | 82.9 |
| Accuracy (%) | 67.9 | 79.2 | 81.9 |  | 84.0 | 89.6 | 83.5 |
| PPV/NPV (%) | 20.6/96.1 | 42.2/94.6 | 29.9/97.7 |  | 36.9/97.5 | 63.3/96.9 | 33.6/98.9 |
| PLR/NLR | 2.30/0.36 | 3.76/0.30 | 4.45/0.25 |  | 5.17/0.23 | 8.89/0.16 | 5.28/0.11 |

Note: F-M: the model established with the indicators of fluorescence intensity; FAHB-M: the model established with the indicators of fluorescence intensity, alpha-fetoprotein, hepatic function tests and blood cell analyses; PHC: primary hepatic carcinoma; BCLC-A/B/C/D: Barcelona Clinic Liver Cancer stage A/B/C/D; NC: normal control; LC: liver cirrhosis; CH: chronic hepatitis; NPHC: non-primary hepatic carcinoma (NC+LC+CH); AUROC: area under the receiver operating characteristic curve; CI: confidence interval; PPV/NPV: positive/negative predictive value; PLR/NLR: positive/negative likelihood ratio.
