# Supplementary material for: Simple and robust diagnosis of early, small and AFP-negative primary hepatic carcinomas: an integrative approach of serum fluorescence and conventional blood tests
Source: Oncotarget. 2016 Aug 31;7(39):64053–70. doi: 10.18632/oncotarget.11771 (PMC5325425; doi:10.18632/oncotarget.11771)
Supplement: Supplementary file 7 [file oncotarget-07-64053-s007.docx]

**Table S6 Diagnostic value of models F-M and FAHB-M for PHC subgroups based on tumor sizes**

| Versus group | F-M | | | | | | |  | FAHB-M | | |
| --- | --- | --- | --- | --- | --- | --- | --- | --- | --- | --- | --- |
|  | PHC tumor size (cm) | | | | | | |  | PHC tumor size (cm) | | |
|  | ≤3(n=67) | | ≤5 (n=109) | | | >5(n=183) | |  | ≤3(n=67) | ≤5(n=109) | >5(n=183) |
| **NC(n=332)** | |  | |  | | | |  |  |  |  |
| AUROC(95%CI) | 0.955(0.927-0.983) | | 0.964(0.943-0.986) | | | 0.960(0.943-0.978) | |  | 0.988(0.974-1.000) | 0.987(0.973-1.000) | 0.997(0.992-1.000) |
| Sensitivity (%) | 82.1 | | 89.9 | | | 88.0 | |  | 92.5 | 94.5 | 98.9 |
| Specificity (%) | 97.3 | | 93.4 | | | 93.7 | |  | 98.8 | 98.8 | 97.9 |
| Accuracy (%) | 94.7 | | 92.5 | | | 91.7 | |  | 97.7 | 97.7 | 98.3 |
| PPV/NPV (%) | 85.9/96.4 | | 81.7/96.6 | | | 88.5/93.4 | |  | 93.9/98.5 | 96.3/98.2 | 96.3/99.4 |
| PLR/NLR | 30.28/0.18 | | 13.57/0.11 | | | 13.91/0.13 | |  | 76.81/0.08 | 78.43/0.06 | 46.91/0.01 |
| **LC(n=331)** | |  | |  | | | |  |  |  |  |
| AUROC(95%CI) | 0.674(0.600-0.748) | | 0.704(0.646-0.761) | | | 0.831(0.795-0.867) | |  | 0.838(0.777-0.899) | 0.863(0.821-0.906) | 0.946(0.927-0.965) |
| Sensitivity (%) | 53.7 | | 61.5 | | | 76.5 | |  | 68.7 | 81.7 | 86.3 |
| Specificity (%) | 76.1 | | 73.4 | | | 75.2 | |  | 88.5 | 79.5 | 90.6 |
| Accuracy (%) | 72.4 | | 70.5 | | | 75.7 | |  | 85.2 | 80.0 | 89.1 |
| PPV/NPV (%) | 31.3/89.0 | | 43.2/85.3 | | | 63.1/85.3 | |  | 54.8/93.3 | 56.7/92.9 | 83.6/92.3 |
| PLR/NLR | 2.25/0.61 | | 2.31/0.52 | | | 3.09/0.31 | |  | 5.98/0.35 | 3.97/0.23 | 9.22/0.15 |
| **CH(n=213)** | |  | |  | | | |  |  |  |  |
| AUROC(95%CI) | 0.761(0.693-0.829) | | 0.758(0.702-0.813) | | | 0.812(0.770-0.853) | |  | 0.935(0.898-0.973) | 0.937(0.908-0.965) | 0.950(0.930-0.969) |
| Sensitivity (%) | 67.2 | | 70.6 | | | 84.2 | |  | 94.0 | 94.5 | 91.8 |
| Specificity (%) | 79.8 | | 71.8 | | | 65.7 | |  | 82.6 | 82.6 | 85.9 |
| Accuracy (%) | 76.8 | | 71.4 | | | 74.2 | |  | 85.4 | 86.6 | 88.6 |
| PPV/NPV (%) | 51.1/88.5 | | 56.2/82.7 | | | 67.8/82.8 | |  | 63.0/97.8 | 73.6/96.7 | 84.8/92.4 |
| PLR/NLR | 3.33/0.41 | | 2.51/0.41 | | | 2.46/0.24 | |  | 5.41/0.07 | 5.44/0.07 | 6.52/0.10 |
| **NPHC(n=876)** | | | | |  | |  |  |  |  |  |
| AUROC(95%CI) | 0.727(0.667-0.786) | | 0.779(0.735-0.824) | | | 0.863(0.832-0.894) | |  | 0.857(0.806-0.907) | 0.884(0.847-0.920) | 0.955(0.940-0.970) |
| Sensitivity (%) | 71.6 | | 74.3 | | | 73.8 | |  | 76.1 | 82.6 | 87.4 |
| Specificity (%) | 62.8 | | 70.0 | | | 85.7 | |  | 84.4 | 84.4 | 89.6 |
| Accuracy (%) | 63.4 | | 70.5 | | | 83.7 | |  | 83.8 | 84.2 | 89.2 |
| PPV/NPV (%) | 12.8/96.7 | | 23.5/95.6 | | | 51.9/94.0 | |  | 27.1/97.9 | 39.6/97.5 | 63.7/97.2 |
| PLR/NLR | 1.93/0.45 | | 2.48/0.37 | | | 5.17/0.31 | |  | 4.87/0.28 | 5.28/0.21 | 8.42/0.14 |

Note: F-M: the model established with the indicators of fluorescence intensity; FAHB-M: the model established with the indicators of fluorescence intensity, alpha-fetoprotein, hepatic function test results and blood cell analyses; PHC: primary hepatic carcinoma; NC: normal control; LC: liver cirrhosis; CH: chronic hepatitis; NPHC: non primary hepatic carcinoma (NC+LC+CH); AUROC: area under the receiver operating characteristic curve; CI: confidence interval; PPV/NPV: positive/negative predictive value; PLR/NLR: positive/negative likelihood ratio.
