# Supplementary material for: Simple and robust diagnosis of early, small and AFP-negative primary hepatic carcinomas: an integrative approach of serum fluorescence and conventional blood tests
Source: Oncotarget. 2016 Aug 31;7(39):64053–70. doi: 10.18632/oncotarget.11771 (PMC5325425; doi:10.18632/oncotarget.11771)
Supplement: Supplementary file 8 [file oncotarget-07-64053-s008.docx]

**Table S7 Comparison of the diagnostic values of models F-M and FAHB-M for PHC diagnosed by pathology and imaging**

| Group compared | F-M | | | | |  | FAHB-M | | |
| --- | --- | --- | --- | --- | --- | --- | --- | --- | --- |
|  | PHC diagnosed by pathology (n=96) | | | | PHC diagnosed by imaging (n=257) |  | PHC diagnosed by pathology (n=96) | | PHC diagnosed by imaging (n=257) |
| **NC (n=332)** | |  | |  | |  |  | |  |
| AUROC(95%CI) | 0.961  (0.938-0.985) | | | | 0.965  (0.950-0.979) |  | 0.994  (0.987-1.000) | | 0.993  (0.986-1.000) |
| Sensitivity (%) | 89.6 | | | | 89.1 |  | 96.9 | | 97.3 |
| Specificity (%) | 93.4 | | | | 93.1 |  | 97.0 | | 98.8 |
| Accuracy (%) | 92.5 | | | | 91.3 |  | 97.0 | | 98.1 |
| PPV/NPV (%) | 79.6/96.9 | | | | 90.9/91.7 |  | 90.3/99.1 | | 98.4/97.9 |
| PLR/NLR | 13.52/0.11 | | | | 12.86/0.12 |  | 32.16/0.03 | | 80.74/0.03 |
| **LC (n=331)** | | |  | | |  |  |  | |
| AUROC(95%CI) | 0.826  (0.778-0.874) | | | | 0.754  (0.715-0.793) |  | 0.935  (0.911-0.960) | | 0.908  0.884-0.933) |
| Sensitivity (%) | 75.0 | | | | 66.9 |  | 85.4 | | 81.7 |
| Specificity (%) | 76.1 | | | | 73.1 |  | 87.6 | | 87.3 |
| Accuracy (%) | 75.9 | | | | 70.4 |  | 87.1 | | 84.9 |
| PPV/NPV (%) | 47.7/91.3 | | | | 65.9/74.0 |  | 66.7/95.4 | | 83.3/86.0 |
| PLR/NLR | 3.14/0.33 | | | | 2.49/0.45 |  | 6.90/0.17 | | 6.44/0.21 |
| **CH (n=213)** | | |  | | |  |  |  | |
| AUROC(95%CI) | 0.799  (0.748-0.850) | | | | 0.798  (0.758-0.839) |  | 0.917  (0.883-0.951) | | 0.955  (0.937-0.973) |
| Sensitivity (%) | 81.3 | | | | 82.1 |  | 90.6 | | 92.2 |
| Specificity (%) | 67.1 | | | | 66.2 |  | 82.6 | | 86.9 |
| Accuracy (%) | 71.5 | | | | 74.9 |  | 85.1 | | 89.8 |
| PPV/NPV (%) | 52.7/88.8 | | | | 74.6/75.4 |  | 70.2/95.1 | | 89.4/90.2 |
| PLR/NLR | 2.47/0.28 | | | | 2.43/0.27 |  | 5.22/0.11 | | 7.02/0.09 |
| **NPH C(n=876)** | | |  | | |  |  |  | |
| AUROC(95%CI) | 0.856  (0.816-0.896) | | | | 0.824  (0.794-0.853) |  | 0.919  (0.890-0.949) | | 0.932  (0.914-0.950) |
| Sensitivity (%) | 82.3 | | | | 70.8 |  | 84.4 | | 87.5 |
| Specificity (%) | 73.4 | | | | 79.7 |  | 86.9 | | 84.4 |
| Accuracy (%) | 74.3 | | | | 77.7 |  | 86.6 | | 85.1 |
| PPV/NPV (%) | 25.3/97.4 | | | | 50.6/90.3 |  | 41.3/98.1 | | 62.2/95.8 |
| PLR/NLR | 3.09/0.24 | | | | 3.49/0.37 |  | 6.43/0.18 | | 5.60/0.15 |
| Note: F-M: the model established with the indicators of fluorescence intensity; FAHB-M: the model established with the indicators of fluorescence intensity, alpha-fetoprotein, hepatic function tests and blood cell analyses; PHC: primary hepatic carcinoma; NC: normal control; LC: liver cirrhosis; CH: chronic hepatitis; NPHC: non primary hepatic carcinoma (NC+LC+CH); AUROC: area under the receiver operating characteristic curve; CI: confidence interval; PPV/NPV: positive/negative predictive value; PLR/NLR: positive/negative likelihood ratio. | | | | | | | | | |
