# Supplementary material for: Simple and robust diagnosis of early, small and AFP-negative primary hepatic carcinomas: an integrative approach of serum fluorescence and conventional blood tests
Source: Oncotarget. 2016 Aug 31;7(39):64053–70. doi: 10.18632/oncotarget.11771 (PMC5325425; doi:10.18632/oncotarget.11771)
Supplement: Supplementary file 9 [file oncotarget-07-64053-s009.docx]

**Table S8 Positive rates of models F-M and FAHB-M and AFP in PHC with different histological types**

|  | Histology | | | Total  (n=353) | P |
| --- | --- | --- | --- | --- | --- |
|  | HCC(n=69) | ICC (n=27) | Unknown(n=257) |  |  |
| Positive cases by F-M (%) | 56(81.2)* | 23(85.2)** | 198(77.0)** | 277(78.5)** | 0.515 |
| Positive cases by FAHB-M (%) | 65(94.2)** | 18(66.7)** | 225(87.5)** | 308(87.3)** | 0.001 |
| Positive cases by AFP (%) |  |  |  |  |  |
| >7 ng/mL | 44(63.8) | 1(3.7) | 157(61.1) | 202(57.2) |  |
| ≥ 20 ng/mL | 29(42.0) | 0(0.0) | 131(51.0) | 160(45.3) |  |
| ≥ 200 ng/mL | 24(34.8) | 0(0.0) | 96(37.4) | 120(34.0) |  |
| ≥ 400 ng/mL | 22(31.9) | 0(0.0) | 91(35.4) | 113(32.0) |  |
| Note: * P<0.05, ** P<0.01, compared with AFP >7 ng/mL. F-M: the model established with the indicators of fluorescence intensity; FAHB-M: the model established with the indicators of fluorescence intensity, alpha-fetoprotein, hepatic function tests and blood cell analyses; HCC: hepatocellular carcinoma; ICC: intrahepatic cholangiocarcinoma; AFP: alpha-fetoprotein. | | | | | |
